# Neural Dynamics of Autistic Repetitive Behaviors and Fragile X Syndrome: Basal Ganglia Movement Gating and mGluR-Modulated Adaptively Timed Learning

**DOI:** 10.3389/fpsyg.2018.00269

**Published:** 2018-03-13

**Authors:** Stephen Grossberg, Devika Kishnan

**Affiliations:** ^1^Center for Adaptive Systems, Graduate Program in Cognitive and Neural Systems, Departments of Mathematics & Statistics, Psychological & Brain Sciences, and Biomedical Engineering, Boston University, Boston, MA, United States; ^2^Department of Biomedical Engineering, Boston University, Boston, MA, United States

**Keywords:** autism, repetitive motor behavior, Fragile X syndrome, mGluR, adaptive resonance theory, spectral timing, basal ganglia, hippocampus

## Abstract

This article develops the iSTART neural model that proposes how specific imbalances in cognitive, emotional, timing, and motor processes that involve brain regions like prefrontal cortex, temporal cortex, amygdala, hypothalamus, hippocampus, and cerebellum may interact together to cause behavioral symptoms of autism. These imbalances include underaroused emotional depression in the amygdala/hypothalamus, learning of hyperspecific recognition categories that help to cause narrowly focused attention in temporal and prefrontal cortices, and breakdowns of adaptively timed motivated attention and motor circuits in the hippocampus and cerebellum. The article expands the model’s explanatory range by, first, explaining recent data about Fragile X syndrome (FXS), mGluR, and trace conditioning; and, second, by explaining distinct causes of stereotyped behaviors in individuals with autism. Some of these stereotyped behaviors, such as an insistence on sameness and circumscribed interests, may result from imbalances in the cognitive and emotional circuits that iSTART models. These behaviors may be ameliorated by operant conditioning methods. Other stereotyped behaviors, such as repetitive motor behaviors, may result from imbalances in how the direct and indirect pathways of the basal ganglia open or close movement gates, respectively. These repetitive behaviors may be ameliorated by drugs that augment D2 dopamine receptor responses or reduce D1 dopamine receptor responses. The article also notes the ubiquitous role of gating by basal ganglia loops in regulating all the functions that iSTART models.

## 1. Introduction

### 1.1 Overview

The core symptoms of autism spectrum disorder have been defined clinically to include deficits in social communication with regards to social reciprocity, communication toward social interaction, and skills required to develop, maintain and understand relationships. Along with the insufficiencies in social communication, the presence of restricted and repetitive patterns of behavior is required for a diagnosis of autism spectrum disorder (American Psychiatric Association, 2013).

**FIGURE 1:**
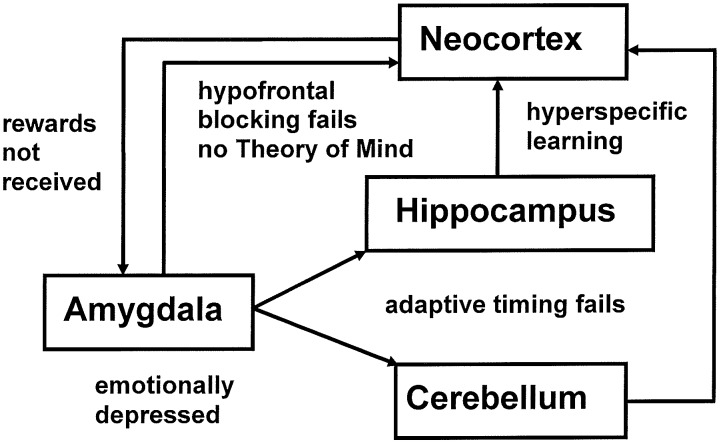
The imbalances in the iSTART model of Grossberg and Seidman (2006) that contribute to autistic behavioral symptoms are: hypervigilance in brain regions like nonspecific thalamus and hippocampus leading to learning of hyperconcrete recognition categories and a narrow focus of attention by brain regions such as the temporal cortex; underarousal of value categories within brain regions such as the amygdala/hypothalamus, leading to elevated thresholds for emotional responsiveness, with the effect of insufficient incentive motivation to support prefrontal processing, but emotional hypersensitivity when these thresholds are exceeded, leading to coping behaviors to avoid these aversive emotions; and an absence of adaptive timing in brain regions such as the hippocampus and cerebellum, leading to attentional distractibility and premature release of actions that typically require delayed activation in order to be socially appropriate. Experimental data that are explained and predicted by these mechanisms are reviewed in Grossberg and Seidman (2006).

The *imbalanced Spectrally Timed Adaptive Resonance Theory*, or iSTART, neural model proposed explanations of symptoms of autism that involve attention, learning, emotion, timing, and social interactions (Grossberg and Seidman, 2006; Grossberg and Vladusich, 2010; Grossberg, 2012). iSTART embodies the same neural mechanisms as the START model, which was used to explain and predict data about the brain mechanisms that learn to control and adaptively time these behaviors in normal individuals (Grossberg and Schmajuk, 1989; Grossberg and Merrill, 1992, 1996), where the word “normal” in the present article refers to “typical” behaviors, as in the normal, or Gaussian, distribution of statistics. In the iSTART model these brain mechanisms become imbalanced in specific ways. Then their emergent properties generate behavioral symptoms of individuals with autism (**Figure 1**). Due to the fact that START and iSTART have the *same* neural mechanisms, a comparison of them clarifies how behavioral symptoms of autism exist on a continuum with typical behavioral properties.

**Figure 1** illustrates multiple brain regions that control different attentional, cognitive, emotional, and timing mechanisms that contribute to behavioral symptoms of autism, in keeping with the fact that autism has been linked to multiple genes (Risch et al., 1999; Lamb et al., 2000; Pickles et al., 2000). The symptoms for which iSTART offered a mechanistic neural interpretation are summarized in **Table 1**.

**Table 1:** Brain processes, their imbalances, and the behavioral symptoms that they cause in the iSTART model, enhanced with the current results about perseverative behaviors due to basal ganglia imbalances.

Brain regions	Imbalance	Symptoms
Non-specific thalamus, hippocampus, cingulate	High vigilance	Hyperconcrete categories, narrow focus of attention
Amygdala, hypothalamus	Underaroused emotional depression	Emotional flatness with emotional hypersensitivity over elevated threshold
Hippocampus	Failure of adaptive spectrally timed learning and performance	Motivated attention cannot be sustained and behaviors that require it not learned or performed
Cerebellum	Failure of adaptive spectrally timed learning and performance	Timed actions cannot be learned or performed
Basal ganglia (SNc)	Failure of adaptive spectrally timed learning and performance	Failure of timed reinforcement learning and performance
Hippocampus, cerebellum, basal ganglia (SNc)	Failure of adaptive spectrally timed learning and performance	Fragile X syndrome
Basal ganglia (SNr)	Increased direct pathway activity and/or decreased indirect pathway activity	Autistic perseverative behaviors


The current article expands the explanatory and predictive range of iSTART. Section 2 describes an important class of data that the iSTART model did not initially try to explain; namely, data concerning mechanistic links between Fragile X syndrome (FXS), metabotropic glutamate receptors (mGluRs), and trace conditioning. Data of this kind will be explained below using already available iSTART mechanisms. Section 2 therefore reviews iSTART mechanisms that are needed to explain Fragile X data in order to provide a self-contained mechanistic neural explanation of these data. These mechanisms include category learning, reinforcement learning, and adaptively timed learning. Their breakdown, notably of hippocampally mediated, mGluR-modulated, adaptively timed learning mechanisms, can lead to Fragile X symptoms.

**FIGURE 2:**
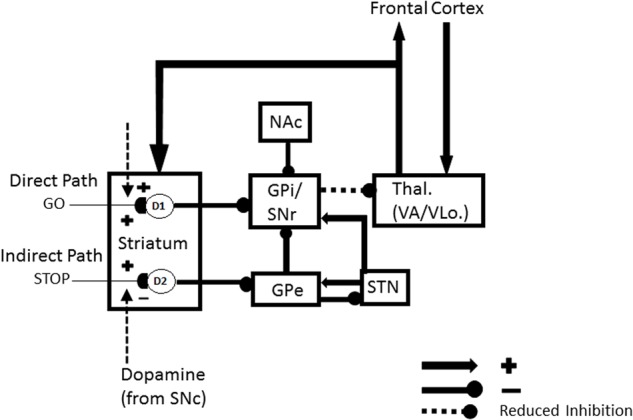
Direct and indirect pathways in the basal ganglia. Excitatory connections end in arrows, inhibitory connections in disks. The basal ganglia direct pathway sends an inhibitory signal from the striatum to the external segment of the globus pallidus (GPi) or the substantia nigra pars reticulate (SNr). The latter regions inhibit the thalamus, and thus the thalamocortical loop. The net effect of inhibiting the GPi/SNr inhibition is to disinhibit the thalamocortical loop. Activation of the direct pathway hereby, at least under normal conditions, acts like a GO signal that enables action plans to be executed. The indirect pathway projects from the striatum to the external segment of the globus pallidus (GPe). The GPe then inhibits the GPi or SNr which, in turn, inhibit the thalamus. Activation of this succession of three inhibitory pathways can inhibit the thalamocortical loop and thereby STOP planned actions, even if there is activation in the direct pathway that would otherwise suffice to generate a GO signal. [Adapted with permission from Brown et al. (2004).] The nucleus accumbens (NAc) can also inhibit the GPi/SNr. Imbalanced activity within the NAc could lead to hyperactivity of the NAc output projection. This increases inhibition of the GPi, which disinhibits the thalamus, thus strengthening the GO signal while neutralizing the impact of the STOP signal. The GO signal can hereby be kept on for a long time, thereby enabling repetitive behavior.

The other kind of data that the article mechanistically explains concern repetitive behaviors in individuals with autism. There are several different kinds of repetitive behaviors, and they may be controlled by different brain regions. These behaviors range from an insistence on sameness (IS) and circumscribed interests (CI) to repetitive motor behaviors (RMB) such as hand clapping and rocking (Lam et al., 2008). The first two kinds of behavior may be explained using iSTART reinforcement learning and motivational mechanisms, and thus may be modified by operant conditioning techniques (Miller and Neuringer, 2000). In contrast, explaining various RMBs requires an extension of the model to include the basal ganglia and its downstream motor control circuits. The basal ganglia *gate* ON and OFF the expression of all kinds of behavior, including perceptual, cognitive, emotional, and motor behaviors. It does this using GO and STOP gates in its direct and indirect pathways, respectively, of the substantia nigra pars reticulate, or SNr (**Figure 2**). How an imbalance in these GO and STOP gates may trigger RMBs will be explained in Section 3.

Section 3 first explains how some repetitive behaviors may be caused by an imbalance in hypothalamic and amygdala opponent processing circuits. Such an imbalance may indirectly lead to an insistence on sameness and circumscribed interest in a manner that will be explained. These are behaviors that can be modified by operant conditioning. Repetitive behaviors may also be caused in individuals who are kept in restricted environments, due to how these environments curtail normal operant behaviors. It is then explained how RMBs, such as movement gaits and saccade staircases, may be caused in normal individuals when basal ganglia gates in the SNr (**Figure 2**), remain open so long that downstream recurrent circuits can persistently oscillate and thereby cause RMBs. Imbalances between the direct and indirect pathways in the basal ganglia of individuals with autism (**Figure 2**) may also cause sustained opening of basal ganglia gates, thereby triggering RMBs that may not be under volitional control. These repetitive behaviors may be ameliorated by treatments that augment D2 dopamine receptor responses or reduce D1 dopamine receptor responses.

### 1.2. A Cyclic Method for Theoretically Linking Mind to Brain

The previous section indicates that the models under consideration undergo incremental, self-consistent refinements in order to explain and predict increasingly large and diverse interdisciplinary databases. The iSTART model and its extensions hereby illustrate a theoretical method that has been successfully developed and applied multiple times during the past 60 years (Grossberg, 1999). This method acknowledges that one cannot “derive an entire brain” all at once.

Because *brain* evolution needs to achieve *behavioral* success, this “method of minimal anatomies” begins with a theoretical analysis of large numbers of *behavioral* experiments. Starting with behavioral data enables the derivation of models whose brain mechanisms have been selected by behavioral success during the evolutionary process. Starting with large numbers of behavioral experiments helps to discard many otherwise seemingly plausible, but wrong, model design principles and mechanisms.

When these design principles and mechanisms are properly embodied in a neural model, the model’s emergent, or interactive, properties help to explain data about how individuals can autonomously learn to adapt in real time to a complex and changing world that is filled with unexpected events. Remarkably, despite being derived from psychological hypotheses, the minimal mathematical models that realize these design principles have always resembled part of a brain.

Mathematical and computational analyses are then used to discover what the minimal model, and its variations, can explain, as well as what it cannot. Such an analysis has always identified additional design principles that the current model does not embody. These new design principles and their mechanistic realizations are then consistently included in the model. These incremental model refinements have gradually led to the current model, which has a much broader interdisciplinary experimental and predictive range than its predecessors. Thus, although a model of the entire brain cannot be derived in one step, the most advanced models that are currently available can individually explain psychological, neurophysiological, neuroanatomical, biophysical, and biochemical data. The current article illustrates this method.

This paper provides a self-contained heuristic overview of relevant computational principles, mechanisms, circuits, and architectures that follow from the above strategy. As noted above, its goal is to provide a parsimonious explanation for symptoms of autistic repetitive behaviors and FXS. Crucially, the sorts of psychopathology that the model accounts for can be understood in terms of imbalances in brain mechanisms that have previously been used to explain and predict large psychological and neurobiological databases about how humans without autism typically learn to attend, recognize, and predict events in a changing world. These explanations thus clarify clinical symptoms and typical behaviors both arise as emergent properties of a shared set of underlying brain designs.

## 2. Fragile X, mGluR, Adaptive Timing, and Trace Conditioning

### 2.1. Neurobiological and Behavioral Data and Model Explanations of Them

First, some data about the relationship between autism and the FXS, which is the most common inherited form of mental retardation (Bear et al., 2004), will be summarized in this section. Then a summary explanation will be given in Sections 2.2 and 2.3 of how such data can be explained by model mechanisms. Finally, the model mechanisms that can explain these data will be described and explained in greater detail in the remainder of this section.

Unlike autism, which may involve symptoms related to multiple genes, FXS is caused by silencing one gene (FMR1) that codes for the Fragile X mental retardation protein (FMRP). FMRP is an RNA-binding protein that is produced in response to activation of group-1 metabotropic glutamate receptors. Belmonte and Bourgeron (2006) note that most cases of autism are not associated with FXS, which has a prevalence of 4% or less. The converse is not, however, true. Estimates of autism in FXS range from 5% to as much as 60%, with recent studies estimating autism in the Fragile X population between 18 and 33%. Most of the difference between autistic and non-autistic FXS subgroups occurs on the social and communicative dimensions of autism, rather than the dimensions of repetitive behaviors and restricted interest. The link between FXS and metabotropic glutamate receptors (mGluRs) has led to “the mGluR theory of Fragile X mental retardation” (Bear et al., 2004).

Children with Fragile X experience severe problems with paying attention (Fryns et al., 1984; Baumgardner et al., 1995) and many are diagnosed with ADHD (Cornish et al., 2004). In order to better understand the neural basis of this deficit, Zhao et al. (2005) developed a mouse model for FXS by knocking out the FMR1 gene. These authors then performed trace conditioning experiments with these mice. Trace conditioning is a form of classical conditioning that associates a neutral event, called the conditioned stimulus (CS), with an emotion-inducing, reflex-triggering event, called the unconditioned stimulus (US). Unlike delay conditioning experiments, wherein the stimulus events temporally overlap, during trace conditioning, a temporal gap separates CS offset and US onset. A CS-activated memory trace must be sustained during the inter-stimulus interval (ISI) in order to learn to associate the CS with the US. Both normal delay and trace conditioning can be accomplished with a range of stimulus durations and ISIs, leading to learning of a conditioned response (CR) that is performed in anticipation of the US.

As the model explains below, the ability to carry out trace conditioning is closely related to the ability to maintain attention upon a task, because the incentive motivation that is sustained during the trace interval helps to maintain motivated attention through time.

In the mouse model for FXS, trace conditioning was severely impaired. This result supports the predicted role in iSTART of metabotropic glutamate receptors (mGluR) in the hippocampally mediated adaptively timed learning that bridges the temporal gap between the CS and US during a trace conditioning experiment. In further support of this prediction, it is known that FMRP is produced at synapses after stimulation of metabotropic glutamate receptors (Weiler and Greenough, 1999), and that metabotropic glutamate receptor-dependent long-term depression is altered in the hippocampus of the FMR1-deficient mice that model the FXS (Huber et al., 2002).

Many experimental and modeling studies have shown an important role of the hippocampus and prefrontal cortex, among other brain regions, in normal and abnormal trace conditioning (Berger et al., 1980; Grossberg and Schmajuk, 1989; Moyer et al., 1990; Sears and Steinmetz, 1990; Grossberg and Merrill, 1992, 1996; Mauk and Ruiz, 1992; Kim et al., 1995; Takehara et al., 2003; Woodruff-Pak and Disterhoft, 2007; Franklin and Grossberg, 2017). In particular, if trace conditioning is followed by a hippocampal lesion, then successful post-acquisition performance of the CR occurs only if the hippocampal lesion occurs after a sufficiently long duration of hippocampal support for memory consolidation within thalamo-cortical and cortico-cortical circuits (Kim et al., 1995; Takehara et al., 2003; Takashima et al., 2009). Indeed, two memory circuits support trace conditioning. One includes the hippocampus and the cerebellum and mediates recently acquired memory, while the other includes the medial prefrontal cortex, or mPFC, and the cerebellum and supports remotely acquired memories (Berger et al., 1986; Takehara et al., 2003).

Additional studies have shown that deletion of FMR1 in cerebellar Purkinje cells causes abnormalities in classical delay eyeblink conditioning, thereby augmenting knowledge about how mGluR and FMR1 abnormalities in cerebral cortical and hippocampal synaptic processes also lead to cognitive, learning, and motor deficits in Fragile X patients (Huber et al., 2002; Koekkoek et al., 2005; Nosyreva and Huber, 2006; Guo et al., 2012; Vinueza Veloz et al., 2012).

### 2.2. Adaptively Timed Learning in Hippocampus, Cerebellum, and Basal Ganglia: Spectral Timing

**FIGURE 3:**
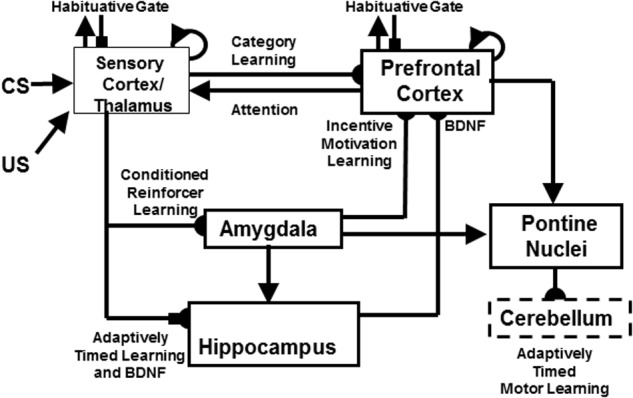
The neurotrophic Spectrally Timed Adaptive Resonance Theory, or nSTART, model macrocircuit is a further development of the START model in which parallel and interconnected networks support both delay and trace conditioing. Connectivity from both the thalamus and the sensory cortex occurs to the amygdala and hippocampus. Sensory cortex interacts reciprocally with the prefrontal cortex, specifically orbitofrontal cortex. Multiple types of learning and neurotrophic mechanisms of memory consolidation cooperate in these circuits to learn and perform adaptively timed responses. Connections from the sensory cortex to the orbitofrontal cortex support category learning. Reciprocal connections from orbitofrontal cortex to sensory cortex support motivated attention. Connections from sensory cortex to amygdala support conditioned reinforcer learning. Connections from amygdala to orbitofrontal cortex support incentive motivation learning. Hippocampal adaptively timed pathways and brain-derived neurotrophic factor (BDNF) bridge temporal delays between CS offset and US onset during trace conditioning acquisition. BDNF also supports long-term memory consolidation within sensory cortex to hippocampal pathways and from hippocampal to orbitofrontal pathways. The pontine nuclei serve as a final common pathway for reading-out conditioned responses. Habituative transmitter gates modulate excitatory conductances at all processing stages in order to prevent uncontrolled persistence of activity due to the positive feedback loops in these circuits. Cerebellar dynamics are not simulated in nSTART. Key: arrowhead = excitatory synapse; hemidisc = adaptive weight; square = habituative transmitter gate; square followed by a hemidisc = habituative transmitter gate followed by an adaptive weight. See text for further details. [Reprinted with permission from Franklin and Grossberg (2017).]

These links between autism, FXS, and mGluR are clarified by the START and iSTART models. The further extension of START to simulate the role of neurotrophins, such as Brain Derive Neurotrophic Factor, or BDNF, in memory consolidation, is called the *neurotrophic START*, or nSTART, model (**Figure 3**; Franklin and Grossberg, 2017). These model variations belong to a still larger family of models that explain and predict how similar neural synaptic and circuit mechanisms for adaptively timed, mGluR-modulated learning seem to operate within the hippocampus, cerebellum, and basal ganglia. As described below, these shared mechanisms enable these different brain regions to carry out different adaptively timed functions.

This shared kind of adaptively timed learning is called *spectrally timed learning* for reasons that are explained in Section 2.6. Spectrally timed learning plays several important roles in the hippocampus, among them to support memory consolidation of learned thalamo-cortical and cortico-cortical recognition categories (**Figure 3**; Franklin and Grossberg, 2017). Hippocampal activity also helps to maintain motivated attentional signals for an adaptively timed duration that enables prefrontal cortical representations to stay active long enough to fully perform the goal-oriented motor responses that they control (Grossberg and Merrill, 1992, 1996). Without this kind of adaptively timed learning, individuals cannot maintain their attention long enough to learn or perform effectively in social settings.

**FIGURE 4:**
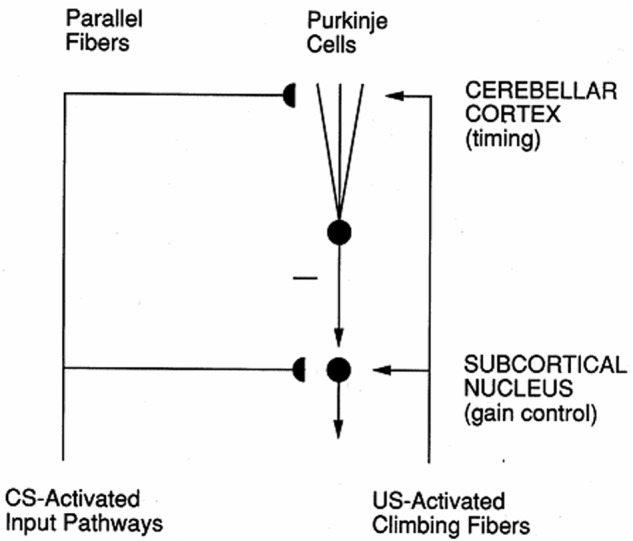
Circuit for adaptively timed cerebellar learning. Adaptively timed Long Term Depression (LTD) at Purkinje cells depresses the level of tonic inhibitory firing of these cells to cerebellar nuclei, thereby disinhibiting cerebellar nuclear cells and allowing them to express their learned gains in an adaptively timed way. LTD occurs when adaptive weights at CS-activated parallel fiber synapses become smaller due to US-activated climbing fiber teaching signals. When this cerebellar circuit interacts with the cortico-hippocampal START or nSTART circuit that controls adaptively timed motivated attention (**Figure 3**), attention can be maintained for an adaptively timed interval that is sufficient to read-out the adaptively timed cerebellar gains that enable an accurate movement to occur. [Reprinted with permission from Grossberg and Merrill (1996).]

In the cerebellum, spectrally timed learning controls adaptively timed motor responses. In particular, it enables adaptively timed Long Term Depression, or LTD, at (parallel fiber)-(Purkinje cell) synapses to disinhibit cerebellar nuclear cells, which can then express learned motor gains that ensure accurate movements in an adaptively timed way (**Figure 4**; Fiala et al., 1996). LTD occurs when CS-activated adaptive weights at the synapses of the parallel fibers are reduced by US-activated teaching signals in the climbing fibers. The hypothesis that mGluR is involved in adaptively timed cerebellar LTD has been supported by subsequent data about calcium signaling and mGluR in the cerebellum (e.g., Finch and Augustine, 1998; Takechi et al., 1998; Ichise et al., 2000; Miyata et al., 2000). Without this kind of adaptively timed learning, individuals cannot learn or perform actions in socially appropriate ways.

**FIGURE 5:**
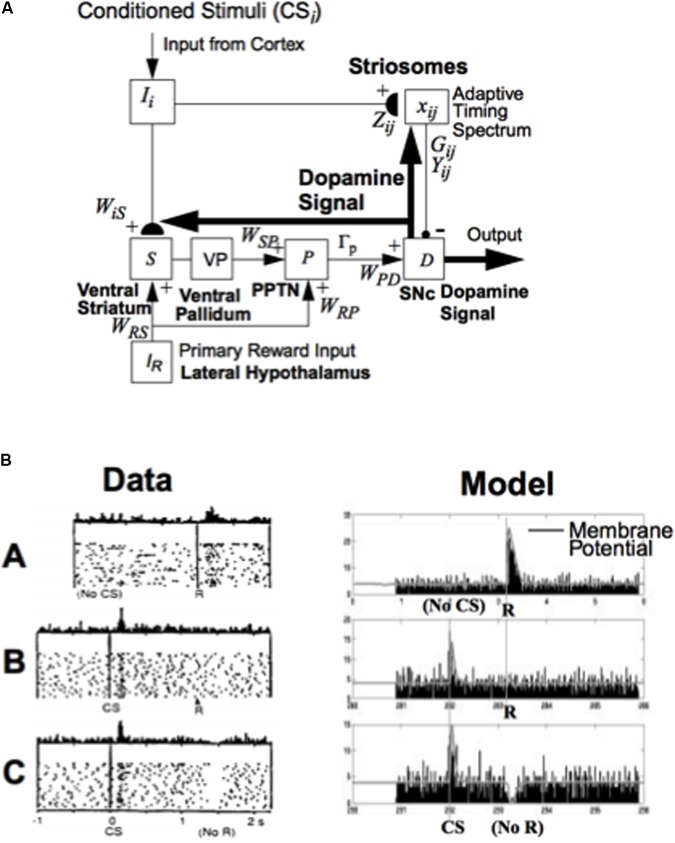
How the basal ganglia generate a dopaminergic Now Print learning signal to multiple brain regions in response to rewards whose timing or amplitude are unexpected: **(A)** Model circuit for triggering dopaminergic Now Print signals at the substantia nigra pars compacta (SNc) to multiple brain regions in response to unexpected rewards. Cortical inputs (I_i_) that are activated by conditioned stimuli learn to excite the SNc (D) via the (ventral striatal, S)-to-(ventral pallidal, VP)-to-(PPTN, P)-to-SNc path. The inputs I_i_ excite the ventral striatum via adaptive weights W_iS_, and the ventral striatum excites the PPTN via double inhibition through the ventral pallidum, with weights W_SP_. When the PPTN activity exceeds a threshold Γ_p_ it excites the dopamine cell with weighted strength W_PD_. The striosomes, which contain an adaptive spectral timing mechanism (x_ij_, G_ij_, Y_ij_, Z_ij_), learn to generate lagged, adaptively timed signals that inhibit reward-related activation of SNc. Primary reward signals (I_R_) from the lateral hypothalamus both excite the PPTN directly (with weighted strength W_RP_) and act as training signals to the ventral striatum S (with weighted strength W_RS_). Arrowheads denote excitatory pathways, circles denote inhibitory pathways, and hemidisks denote synapses at which learning occurs. Thick pathways denote dopaminergic signals. **(B)** Dopamine cell firing patterns: Left: Data. Right: Model simulation, showing model spikes and underlying membrane potential. (A) In naive monkeys, the dopamine cells fire a phasic burst when unpredicted primary reward R occurs; e.g., if the monkey receives a burst of apple juice unexpectedly. (B) As the animal learns to expect the apple juice that reliably follows a conditioned stimulus (CS) that precedes it by a fixed time interval, then the phasic dopamine burst disappears at the expected time of reward, and a new burst appears at the time of the reward-predicting CS. (C) After learning, if the animal fails to receive reward at the expected time, a phasic depression in dopamine cell firing occurs. Thus, these cells reflect an adaptively timed expectation of reward that cancels the expected reward at the expected time. [The data in **(B)** (column 1) are reprinted with permission from Schultz et al. (1997)]. [The model diagram in **(A)** and data simulation in **(B)** (column 2) are reprinted with permission from Brown et al. (1999).]

**FIGURE 6:**
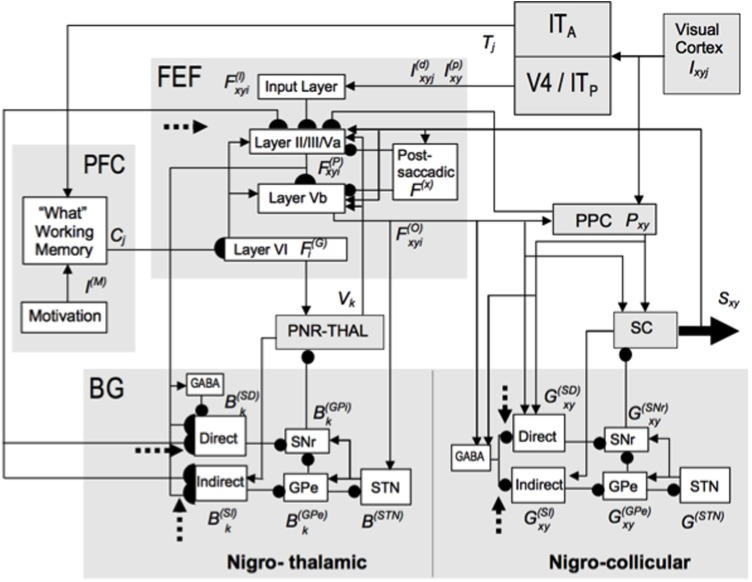
The TELOS (TElencephalic Laminar Objective Selector) neural model of how basal ganglia (SNr) interactions gate learning of saccadic eye movement strategies. Due to the fact that parallel basal ganglia circuits regulate all aspects of cognition and behavior, similar basal ganglia dynamics may be expected in the explanations of many types of cognition and behavior. Separate gray-shaded blocks highlight the major anatomical regions whose roles in planned and reactive saccade generation are treated in the model. Excitatory links are shown as arrowheads, inhibitory as ballheads. Filled semi-circles terminate cortico-striatal and corticocortical pathways modeled as subject to learning, which is modulated by reinforcement-related dopaminergic signals (dashed arrows). See the archival article for details. [Reprinted with permission from Brown et al. (2004).]

In the basal ganglia, spectrally timed learning enables the substantia nigra pars compacta (SNc) to generate widespread Now Print dopaminergic learning signals in response to unexpected reward (**Figure 5**; Brown et al., 1999). A Now Print learning signal is a signal that is broadcast broadly to many brain regions where it can modulate learning at all of its recipient neurons (Livingston, 1967; Grossberg, 1974; McGaugh, 2003; Harley, 2004). These Now Print signals support learning of new associative links between different brain regions during reinforcement learning; e.g., between the posterior part of the inferotemporal cortex (ITp) and the frontal eye fields (FEF) when learning to control saccadic eye movements to visually presented targets (**Figure 6**; Brown et al., 2004). Without this kind of adaptively timed learning, individuals cannot learn from changing reinforcement schedules, and so cannot effectively adapt to the flux of changing social contingencies.

A failure of any or all of these adaptively timed learning circuits could cause problems in both Fragile X individuals and individuals with autism. Consistent with this conclusion, it is known that some individuals with autism fail to exhibit adaptively timed responses when they are tested in various learning paradigms; e.g., Sears et al. (1994) and Szelag et al. (2004). See Grossberg and Seidman (2006) for a more extensive data review of autistic symptoms.

### 2.3. Explaining Fragile X Symptoms

Significantly, these spectral timing model explanations of how mGluR may influence adaptively timed learning and its consequences for various types of behavior (e.g., Fiala et al., 1996) preceded much of the data showing a role for mGluR in FXS. Thus, Fragile X symptoms that are explained by the model may be viewed as confirmed predictions of the model.

All the main Fragile X symptoms have such a mechanistic explanation. For example, children with Fragile X experience behavioral problems of severe inattention (Fryns et al., 1984; Baumgardner et al., 1995) and ADHD symptoms (Cornish et al., 2004) because their hippocampal adaptively timed circuits cannot maintain motivated attention long enough to successfully carry out many behaviors. A mouse model for FXS experiences severe impairment of trace conditioning (Zhao et al., 2005) because its circuit for spectral timing in the hippocampus is not working. Finally, mGluR and FMR1 abnormalities in cerebral cortical and hippocampal synaptic processes cause deficient cognitive, learning, and motor deficits in Fragile X patients (Huber et al., 2002; Koekkoek et al., 2005; Nosyreva and Huber, 2006; Guo et al., 2012; Vinueza Veloz et al., 2012) because of the several ways, summarized above, in which hippocampal, basal ganglia, cerebellar, and basal ganglia circuits can break down if their mGluR-supported spectrally timed circuits are not working.

The next sections review key modeling concepts and mechanisms about the neural learning and information processing mechanisms that are needed to more deeply understand how Fragile X symptoms are caused, and how some symptoms of individuals with autism are caused. This review summarizes how objects and events are recognized, and how these recognized events activate emotions, motivated attention, and goal-oriented actions in an adaptively timed way. All of these processes interact within *recurrent* neural networks in which feedback between the processes influences each of their properties, as in the nSTART circuit in **Figure 3**.

### 2.4. ART Resonance and Reset Control Category Learning and Memory Search

All sufficiently advanced brains solve the *stability-plasticity dilemma* (Grossberg, 1980). This dilemma concerns how individuals can quickly learn to attend, recognize, and predict new objects and events, without that new learning causing catastrophic forgetting of previously learned memories. Adaptive Resonance Theory, or ART, proposes how this problem is solved (Grossberg, 1976, 1980; Carpenter and Grossberg, 1987, 1991) using matching between bottom-up input patterns and learned top-down expectations at networks of feature-selective cells. As reviewed in Grossberg (2013, 2017b), all the main predictions about ART design principles and mechanisms have been supported by both psychological and neurobiological data.

**FIGURE 7:**
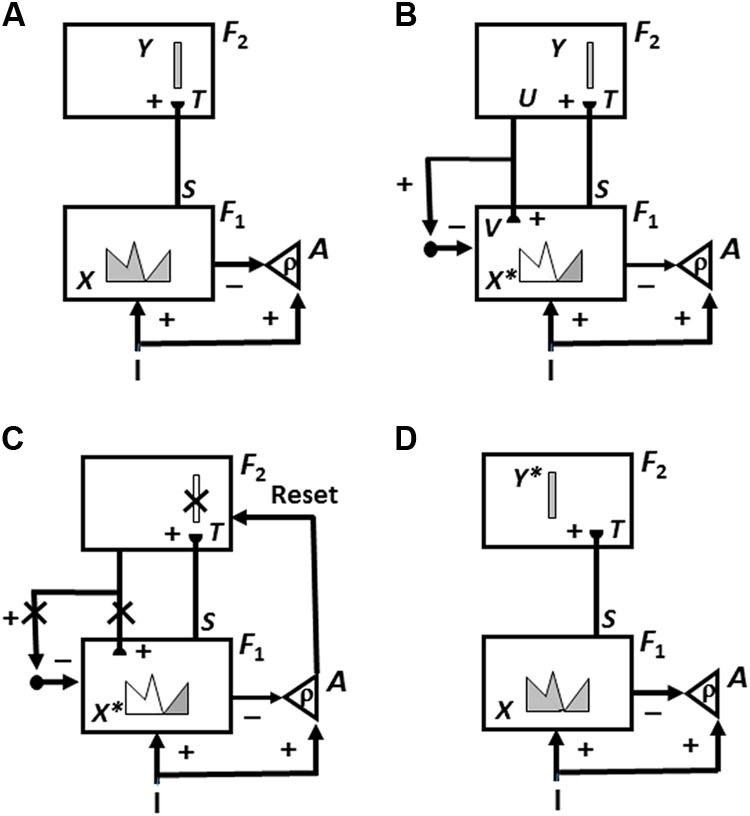
Cycles of ART memory search and category learning using the ART Matching Rule. ART searches for and learns a new recognition category using cycles of match-induced resonance and mismatch-induced reset. Active cells are shaded gray; inhibited cells are not shaded. **(A)** Input pattern *I* is instated across feature detectors at level *F*_1_ as an activity pattern *X*, at the same time that it generates excitatory signals to the orienting system *A* with a gain *ρ* that is called the *vigilance* parameter. Activity pattern *X* generates inhibitory signals to the orienting system *A* as it generates a bottom-up input pattern *S* to the category level *F*_2_. A dynamic balance within *A* between excitatory inputs from *I* and inhibitory inputs from *S* keeps *A* quiet. The bottom-up signals in *S* are multiplied by learned adaptive weights to form the input pattern *T* to *F*_2_. The inputs *T* are contrast-enhanced and normalized within *F*_2_ by recurrent lateral inhibitory signals that obey the membrane equations of neurophysiology, otherwise called shunting interactions. This competition leads to selection and activation of a small number of cells within *F*_2_ that receive the largest inputs. In this figure, a winner-take-all category is chosen, represented by a single cell (population). The chosen cells represent the category Y that codes for the feature pattern at *F*_1_. **(B)** The category activity *Y* generates top–down signals *U* that are multiplied by adaptive weights to form a prototype, or critical feature pattern, *V* that encodes the expectation that the active *F*_2_ category has learned for what feature pattern to expect at *F*_1_. This top–down expectation input *V* is added at *F*_1_ cells using the ART Matching Rule, whereby object attention activates a top–down, modulatory on-center, off-surround network. The on-center of *V* selects features that match it while synchronizing and gain-amplifying them. The off-surround suppressed mismatched features. In other words, due to the off-surround, features of *I* that mismatch *V* at *F*_1_ are inhibited, leading to a new STM activity pattern *X^∗^* within cells whose activities match *V* well enough in its on-center [the gray pattern in **(B)** and **(C)**]. In other words, *X^∗^* is active at *I* features that are confirmed by *V*. Mismatched features (white area) are inhibited. When *X* changes to *X^∗^*, total inhibition decreases from *F*_1_ to *A*. **(C)** If inhibition decreases sufficiently, *A r*eleases a nonspecific arousal burst to *F*_2_; that is, “novel events are arousing.” Within the orienting system *A*, a vigilance parameter ρ determines how bad a match will be tolerated before a burst of nonspecific arousal is triggered. This arousal burst triggers a memory search for a better-matching category, as follows: Arousal resets *F*_2_ by inhibiting *Y.* **(D)** After *Y* is inhibited, *X* is reinstated and *Y* stays inhibited as *X* activates a different category, that is represented by a different activity winner-take-all category *Y^∗^*, at *F*_2._. Search continues until a better matching, or novel, category is selected. When search ends, an attentive resonance triggers learning of the attended data in adaptive weights within both the bottom–up and top–down pathways. As learning stabilizes, inputs *I* can activate their globally best-matching categories directly through the adaptive filter, without activating the orienting system. [Adapted with permission from Carpenter and Grossberg (1987).]

Category learning in ART is controlled by cycles of resonance and reset that are regulated by interactions between an attentional system and an orienting system that obey computationally *complementary* laws (**Figure 7**; Grossberg, 1980, 2000a, 2013, 2017b). The attentional system carries out processes like attention, category learning, expectation, and resonance when there is a good enough match between bottom–up feature patterns and top–down expectations. Object attention in ART obeys an ART Matching Rule that is realized by a top–down, modulatory on-center, off-surround network whose predicted properties have been supported by many subsequent psychological and neurobiological experiments (see Grossberg, 2013, 2017b for reviews.) When a sufficiently bad mismatch occurs between this top–down attentive network and a bottom–up input pattern, the orienting system is activated and resets the attentional system, thereby leading to a memory search, or hypothesis testing, that automatically discovers a category that can learn to better represent incoming bottom-up input patterns (**Figure 7**). The orienting system enables the attentional system to rapidly learn about novel information without experiencing catastrophic forgetting. The attentional system includes brain regions like the temporal cortex and prefrontal cortex. The orienting system includes brain regions like the non-specific thalamus and hippocampus.

#### Vigilance Control Determines How General or Concrete Learned Categories Will Be

How good a match is required for resonance and category learning to occur? The answer to this question clarifies how some of the most familiar cognitive symptoms of autism arise.

The matching criterion is set by a *vigilance parameter* ρ that is computed within the orienting system *A* (**Figure 7**; Carpenter and Grossberg, 1987, 1993). The size of the vigilance parameter determines the generality of the recognition categories that will be learned. If vigilance is high, then learning of a concrete or specific category occurs, such as learning to recognize a frontal view of a familiar face. If vigilance is low, then learning of an abstract or general category occurs, such as learning to recognize that everyone has a face. In general, vigilance is chosen as low as possible to conserve memory resources, without causing a reduction in predictive success. Because baseline vigilance level is initially set at the lowest level that has led to predictive success in the past, ART models try to learn the most general categories that are consistent with their experiences. This property may clarify the overgeneralization that occurs in young children (Brooks et al., 1999) until category refinement is achieved by subsequent learning (Tomasello and Herron, 1988).

When a given task requires a finer categorization, vigilance is raised. Vigilance can be automatically adjusted to learn either concrete or general information in response to predictive failures, or disconfirmations, within each environment. Such a predictive failure could occur, for example, if a viewer classifies an object as a dog, whereas it is really a fox. Within ART, such a predictive disconfirmation causes a memory search that automatically shifts attention to focus on a different combination of features that can successfully be used to learn and subsequently recognize that the object is, in fact, a fox.

One way that vigilance can change due to a predictive error is by a process of *match tracking*. Here, vigilance is increased in response to a predictive error by the minimum amount that is needed to drive a search for a more predictive category (Carpenter and Grossberg, 1987). Since lower vigilance allows learning of more general categories, match tracking learns predictive categories by sacrificing the minimum amount of category generality. It hereby realizes a kind of *minimax learning* that conjointly maximizes generalization while minimizing predictive error.

A great deal is now known about how vigilance is computed in the brain. For example, a sufficiently big mismatch due to a predictive disconfirmation can activate the nucleus basalis of Meynert which, in turn, can release acetylcholine (ACh) at cortical layer 5 cells. ACh can then trigger a search for a better matching category, even if the previous match was deemed sufficient. Many challenging psychological and neurobiological data about cortical regulation of category learning and memory that can be explained by this vigilance mechanism are described in Grossberg and Versace (2008), Palma et al. (2012a,b), and Grossberg (2017a). In particular, a catastrophic collapse of both tonic and phasic vigilance control can help to explain how the dynamics of learning, recognition, and cognition fail during Alzheimer’s disease, and why disorders such as Alzheimer’s disease and autism are often accompanied by abnormal sleep patterns (Grossberg, 2017a). Also modeled are how these ART dynamics can be incorporated into larger neural architectures that are capable of learning view-, size-, and position-invariant object categories and using them to search for desired objects in a cluttered scene (Cao et al., 2011; Grossberg et al., 2011; Chang et al., 2014; Grossberg, 2018).

#### High Vigilance, Hyperspecific Category Learning, and Attentional Deficits in Autism

High vigilance has been predicted to cause symptoms of hyperspecific category learning and attentional deficits in some individuals with autism (Grossberg and Seidman, 2006). This prediction has been successfully tested in psychophysical experiments showing that hyperspecific category learning occurs in high functioning individuals with autism (Church et al., 2010; Vladusich et al., 2010), thereby augmenting previous reports of problems with prototype learning in individuals with autism (e.g., Klinger and Dawson, 2001). It is also known that individuals with autism can exhibit abnormal cholinergic activity in the parietal and frontal cortices that correlates with nucleus basalis abnormalities (Perry et al., 2001), as well as neuron pathology (Kemper and Bauman, 1998) and morphological abnormalities (Riva et al., 2011), consistent with our account of how vigilance is controlled by the nucleus basalis via ACh release.

Hypervigilance can have multiple effects on learning and cognition. In particular, variations in social situations that might otherwise be categorized as familiar can lead to many resets and attention shifts in a hypervigilant individual, thereby preventing effective learning and performance in them. Section 3 will clarify how, when hypervigilance interacts with underaroused emotional depression in an individual with autism, highly aversive emotional responses may be triggered, whose avoidance may lead to coping strategies that include an insistence on sameness and circumscribed interests.

Bayesian models of autism include a concept of *precision* that may be compared and contrasted with the ART concept of vigilance. For example, the Lawson et al. (2014) article about their “aberrant prediction account of autism” states that “The discrepancy between the sensory input and descending predictions of that input is known as the *prediction error*. This prediction error reports what stimulus-associated information is ‘newsworthy’ in the sense that it was unpredicted and informative. This information is *passed up the hierarchy to inform higher-level expectations, which subsequently generate better predictions and thereby resolve prediction errors*. The influence of (top-down) prior beliefs, relative to (bottom-up) sensory evidence, is controlled by the *precision*, or confidence placed in prediction errors at each level of the hierarchy (Friston, 2008). A high sensory precision will *increase the influence of ascending prediction errors by turning up the ‘volume’ of sensory channels in which we place more confidence*…*Crucially, if the predictive coding account on offer is true, precision itself has to be estimated, much like estimating a standard error in statistics, in terms of its expectation*…” [italics ours].

Although every prediction theory needs to somehow cope with fine vs. coarse predictions, the above Bayesian account differs in multiple ways from ART in terms of both heuristics and mechanisms. For example, the ART Matching Rule does not a compute a “prediction error” that is “passed up the hierarchy to inform higher-level expectations, which subsequently generate better predictions and thereby resolve prediction errors.” Instead, the ART Matching Rule uses excitatory matching to generate resonant brain states that trigger learning, and big enough mismatches to drive a memory search to discover categories whose critical feature patterns, or prototypes, will learn to better represent the current input pattern, without requiring a hierarchy of “higher-level expectations.”

ART does not require “higher-level expectations” because it uses *computationally complementary* attentional and orienting parallel processing systems, which have detailed support from multiple kinds of experiments. Vigilance could not be defined, or trigger a memory search, without interactions between these attentional and orienting streams. Vigilance does not have “to be estimated, much like estimating a standard error in statistics, in terms of its expectation.” Rather, vigilance just determines when an input exemplar is too novel to be classified by a previously learned category—for multiple possible reasons, emotional, cognitive, cultural—and drives a search process that automatically discovers and learns a more predictive category. When vigilance control carries out match tracking, it automatically realizes a kind of minimax learning in response to any sufficiently big mismatch, without any explicit link from the expectation that caused the mismatch to vigilance change.

It here needs to be kept in mind that the prediction that is mismatched in the world is not the top–down expectation that is learned to dynamically stabilize the learning of the category itself, and these expectations can represent totally different things; e.g., motor outcomes vs. sensory categories. Indeed, ART has been derived from a thought experiment that shows how the need to overcome several kinds of uncertainty leads directly to ART mechanisms (Grossberg, 1980). Finally, these ART concepts and mechanisms have successfully explained and simulated many psychological and neurobiological data, and all the main ART predictions have been supported by such data. In contrast, a Bayesian account does not have a natural representation in terms of identified brain circuits and regions. Nor does it represent the real-time interactive dynamics whereby brains give rise to the emergent properties of observable behaviors.

### 2.5. CogEM Reinforcement Learning, Motivated Attention, and Directed Action to Valued Goals

**FIGURE 8:**
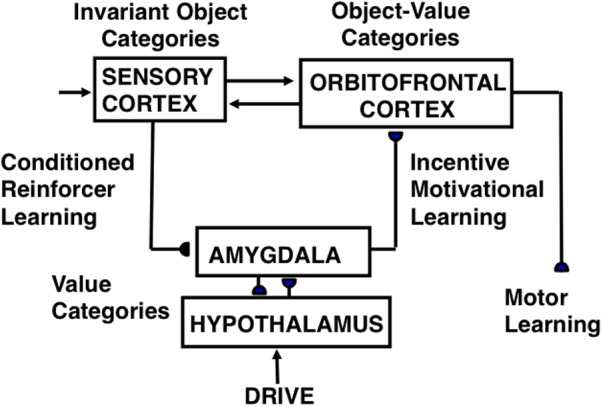
CogEM (Cognitive-Emotional Motor) neural model circuits and their anatomical interpretation. The two successive stages of a sensory representation are interpreted to be invariant object categories in a sensory cortex—such as inferotemporal cortex in the case of vision—and object-value categories in its orbitofrontal projection. An object-value category requires incentive motivational support from a value category in the amygdala/hypothalamus, in addition to an input from its invariant object category, to fire vigorously and win a competition among other object-value categories. The winning object-value category then sends positive feedback to its companion invariant object category representation in sensory cortex, thereby selectively amplifying and focusing motivated attention upon motivationally relevant sensory events, and attentionally blocking other events. Three types of learning occur in the CogEM model, conditioned reinforcer learning, incentive motivational learning, and motor learning. [Adapted with permission from Grossberg and Seidman (2006).]

These ART invariant recognition categories represent external information about the world, but do not evaluate how important this information is for survival or success. Interactions between perceptual/cognitive and evaluative reinforcement/emotional/motivational mechanisms accomplish this. The Cognitive-Emotional-Motor (CogEM) model (**Figure 8**) and its variants propose how emotional centers of the brain, such as the amygdala and hypothalamus, interact with the sensory and prefrontal cortices to undergo reinforcement learning and to thereby support motivated behaviors (Grossberg, 1971, 1972a,b, 1982, 2000b; Grossberg and Levine, 1987; Grossberg and Schmajuk, 1987; Grossberg and Seidman, 2006).

After reinforcement learning occurs, the amygdala can focus motivated attention on recognition categories whose activation generate actions that can acquire valued reward. However, the amygdala cannot, by itself, *maintain motivated attention during an adaptively timed interval* so that reward that are delayed in time can be acquired. The hippocampus is needed to do this, as Section “Adaptively Timed Conditioning and Behavior and Its Breakdown during Fragile X” will explain.

#### Cognitive-Emotional Resonance: Conscious Feelings, Motivated Attention, and Action

The CogEM model (**Figure 8**) explains how object categories, in sensory cortical regions like ITa, and object-value categories, in cortical regions like orbitofrontal cortex, interact with value categories, in subcortical emotional centers like amygdala and hypothalamus. These brain regions are linked by a feedback loop which, when activated for sufficiently long time, can generate a *cognitive-emotional resonance*. Such a resonance can support conscious feelings while using conditioned reinforcer pathways (from sensory cortex to amygdala) and incentive motivation pathways (from amygdala to orbitofrontal cortex) to focus motivated attention upon valued object representations. These attended object representations can, in turn, release commands to perform actions compatible with these feelings. The next two sections say more about these several types of categories and the learned interactions between them.

#### Object, Value, and Object-Value Categories

Four different types of learned representations are included in the CogEM circuit of **Figure 8**: *Invariant object categories* respond selectively to objects that are seen from any perspective. As noted above, they occur in ITa, among other cortical regions. *Value categories* are sites of reinforcement learning that control different emotions and incentive motivational output signals. They occur in amygdala and hypothalamus. *Object-value categories* respond to converging signals from object and value categories. They occur in orbitofrontal cortex. Finally, *motor representations* (M) control motor actions. They occur in multiple brain regions, including motor cortex and cerebellum.

#### Three Kinds of Learning: Reinforcement, Incentive Motivational, and Motor Learning

Three types of learning are shown in **Figure 8** between these representations: *Conditioned reinforcer learning* strengthens the pathway from an invariant object category to a value category. *Incentive motivational learning* strengthens the pathway from a value category to an object-value category. *Motor learning* enables the performance of an act aimed at acquiring a valued goal object. A fourth kind of learning strengthens the connections between an invariant object category and its object-value category during memory consolidation. This last kind of learning will not be further explained here. It is included in the nSTART circuit (**Figure 3**) that augments CogEM to include both a type of adaptively timed learning, called *spectral timing*, that involves the hippocampus, and modulation of memory consolidation by brain-derived neurotrophic factor, or BDNF (Franklin and Grossberg, 2017). When all of these factors interact within nSTART, the model can explain and simulate the complex pattern of memory consolidation problems that arises if learning is followed by early vs. late ablations of amygdala, hippocampus, or orbitofrontal cortex, including symptoms of the famous amnesic patient HM (Milner et al., 1968). Section “Adaptively Timed Conditioning and Behavior and Its Breakdown during Fragile X” summarizes the relevance of breakdowns in spectral timing toward explaining symptoms of FXS.

Reinforcement learning, say classical conditioning (Pavlov, 1927/1960; Kamin, 1968, 1969), occurs within conditioned reinforcer pathways (**Figure 8**) that convert a CS into a *conditioned reinforcer* when its object category is activated sufficiently often just before the value category is activated by an US, or other previously conditioned reinforcer CSs. As a result of this kind of learning, a CS can subsequently activate a value category via this learned pathway. When this happens, the CS is said to be a *conditioned reinforcer* because it can cause many of the same reinforcing and emotional effects as a US.

During classical conditioning, incentive motivational learning also occurs from the activated value category to the object-value category that corresponds to the CS, Incentive motivational learning enables an active value category to prime, or modulate, the object-value categories of all CSs that have consistently been correlated with it. It is the kind of learning that enables you to think of favorite foods when you are hungry.

Motor, or habit, learning adaptively calibrates sensorimotor maps, vectors, and gains that are used for sensory-motor control, after which a CS can read-out correctly calibrated movements via its object-value category.

Although the above summary describes only classical conditioning, the CogEM model was, in fact, introduced to explain key data about operant conditioning (Grossberg, 1971). Many reinforcement learning and motivated attentional mechanisms exploit shared neural circuits, even though the experimental paradigms and behaviors that activate these circuits may differ.

#### Polyvalent Constraints on Cell Firing Ensure That Only Valued Actions Are Triggered

The CogEM circuit in **Figure 8** needs to have two successive sensory processing stages, an invariant object category stage in the temporal cortex, and an object-value category stage in orbitofrontal cortex, in order to ensure that the object-value category can release motivated behavior only if both sensory and motivational support for that behavior is provided as inputs to the object-value category. A *polyvalent constraint* on an object-value category prevents it from firing unless it simultaneously receives input from its invariant object category *and* from a value category. In other words, an object-value category can fire only when the action that it controls is valued at that time. Only when it fires can an object-value category trigger an action. After learning occurs, a conditioned reinforcer can satisfy the polyvalent constraint by sending a signal directly to its object-value category, and indirectly to the object-value category via the (conditioned reinforcer)-(incentive motivational) pathway.

Each value category in the amygdala/hypothalamus also obeys a polyvalent constraint because it also needs two converging inputs in order to fire: a reinforcing input from a US or conditioned reinforcer CS *and* a sufficiently large internal drive input (e.g., hunger, thirst). Each value category can only then generate large incentive motivational output signals to object-value categories.

Thus, both the value categories and the object-value categories obey polyvalent constraints: Due to these constraints, a reinforcing cue does not activate strong incentive motivation, and with it action, to satisfy a drive that is already satisfied.

#### The Feeling of What Happens and the Somatic Marker Hypothesis

Previous articles review some of the many psychological and neurobiological data that the CogEM model has explained and predicted, and how it compares with other models of cognitive-emotional dynamics; e.g., Grossberg (2013, 2017a,b, 2018). One particularly interesting comparison relates to the ability of the CogEM model to explain and predict clinical data. Damasio (1999) has derived from clinical data a heuristic version of the CogEM model, and used it to describe cognitive–emotional resonances that support “the feeling of what happens.” Each processing stage in Damasio’s model (see his **Figure 6**) corresponds to a processing stage in the CogEM circuit of **Figure 8**. In particular, the “map of object X” corresponds to the sensory cortical stage where invariant object categories are represented. The “map of the proto-self” becomes the value category and its multiple interactions. The “second-order map” becomes the object–value category. And the “map of object X enhanced” becomes the object category as it is attentively amplified by feedback from the object–value category. As this cognitive–emotional resonance develops through the excitatory feedback loop between object, value, and object–value categories, the attended object achieves emotional and motivational significance, and motivated decisions are made that can trigger context-appropriate actions toward valued goals.

CogEM hereby embodies, and anticipated, key concepts of the “somatic marker hypothesis” which proposes that decision-making depends upon emotion, while also providing a mechanistic neural explanation (e.g., Grossberg et al., 2008) of the different properties of amygdala and orbitofrontal cortex in making these decisions (Bechara et al., 1999; Baxter et al., 2000; Schoenbaum et al., 2003). In particular, the effects of amygdala or orbitofrontal lesions on subsequent behaviors are described and explained in Grossberg et al. (2008) and Grossberg (2018), including how such lesions influence the brain’s computation of an object’s “desirability” (Rudebeck et al., 2017).

### 2.6. Adaptively Timed Conditioning and Behavior and Its Breakdown During Fragile X

Terrestrial animals are able to avoid the grim fate of restlessly exploring the world for immediate gratifications until prematurely dying. One way that they do this is by learning to time their behaviors to acquire delayed rewards. How delays in reinforcement influence learning and behavior is studied using laboratory paradigms such as trace conditioning and delayed non-match to sample.

The CogEM model cannot learn from temporally delayed reward and punishments, and cannot learn to adaptively time behaviorally responses that need to bridge a temporal delay. The START model, which includes all CogEM processes, can do so by also incorporating adaptively timed learning circuits in the hippocampus and cerebellum. The nSTART model (**Figure 3**) also includes these circuits (Franklin and Grossberg, 2017). As the following sections summarize, these adaptively timed learning mechanisms enable trace conditioning to occur, and are predicted to be the mGluR-dependent processes that break down during FXS.

#### Expected vs. Unexpected Non-occurrences of Reinforcing Events

Many terrestrial animals learn to time their behaviors by distinguishing *expected disconfirmations (or non-occurrences)* of reward from *UNexpected disconfirmations* (or *non-occurrences*) of reward (Grossberg and Schmajuk, 1989; Grossberg and Merrill, 1992, 1996). An *expected* non-occurrence is said to occur if a reward is expected roughly a fixed amount of time after a discriminative cue occurs in a given situation. The non-occurrence of the reward before that time is then not interpreted as a predictive failure. Such an expected non-occurrence does not lead to a reset of short-term memory, an attention shift to focus on other events, emotional frustration, and/or the release of exploratory behaviors to enable search for the desired goal object elsewhere. If, however, the reward does not occur at the expected time, and is thus an *unexpected* non-occurrence, then these cognitive, attentional, emotional, and motor consequences can occur to enable the animal to find the desired goal object elsewhere.

**FIGURE 9:**
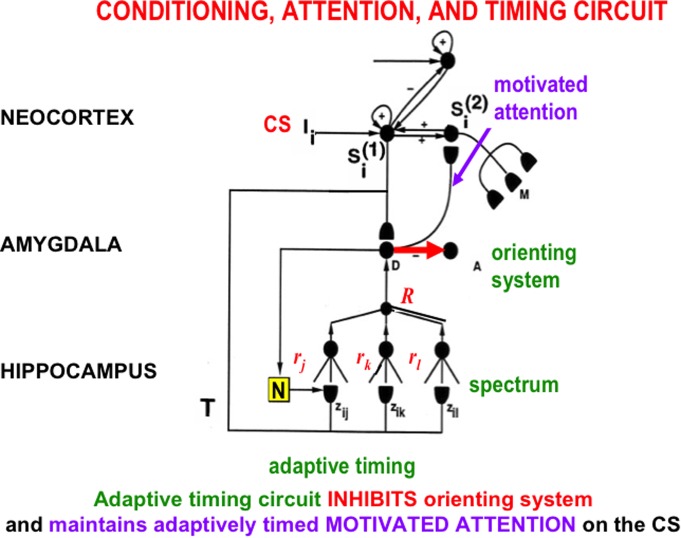
The simplest version of the START (Spectrally Timed Adaptive Resonance Theory) neural model. Adaptively timed learning maintains motivated attention within the temporal-amygdala-orbitofrontal feedback loop (**Figure 8**) at the same time that it inhibits activation of the orienting system. START hereby combines the reinforcement learning, motivated attention, and action processes of the CogEM model with adaptively timed inhibition of the ART orienting system *A* (**Figure 7**). A sensory cortical representation *S_i_*^(1)^ is activated by the CS input *I_i_*, and then tries to activate its orbitofrontal cortical projection in *S_i_*^(2)^. This happens while it also competes with other sensory representations and sends conditioned reinforcer signals to the drive representation, D, which plays the role of the amygdala in the model. Learning from *S_i_*^(1)^ to D is conditioned reinforcer learning, whereas learning from D to *S_i_*^(2)^ is incentive motivational learning. The *S_i_*^(1)^-to-*D*-to-*S_i_*^(2)^-to-*S_i_*^(1)^ feedback loops maintain motivated attention upon motivationally salient objects and events. A parallel branch from the sensory cortex *S_i_*^(1)^ goes to the hippocampus where a spectrum of cells responds at different rates (r_j_, r_k_, r_l_) to the input signal T (see **Figure 10**). The population response of these cells supports correctly timed learning that can bridge the temporal gaps that occur during trace conditioning and delayed non-match to sample, among other paradigms. When the adaptively timed circuit is active, it maintains motivated attention via the feedback pathway (pathway *D* → *S_i_*^(2)^ → *S_i_*^(1)^ → *D*) for an adaptively timed interval, while it inhibits activation of the orienting system (pathway *D* → *A*) in order to prevent distracting events from interfering with the adaptively timed response that is read out by *S_i_*^(2)^ to acquire a valued goal. [Adapted with permission from Grossberg and Merrill (1992).]

The START model explains how hippocampal activity can *maintain motivated attention* when an expected non-occurrence occurs, via a learned hippocampal-to-orbitofrontal incentive motivational pathway (**Figure 3**), while it also *inhibits the orienting system A* (**Figure 9**). This is the same orienting system that, left uninhibited, would otherwise cause a reset of short-term memory, a shift of attention, emotional frustration, and/or the release of exploratory behaviors as part of the ART category learning and memory search circuit (**Figure 7C**). How frustration can be triggered by an unexpected event is explained in Section 3.

#### Social Consequences of a Failure of Adaptively Timed Learning

An animal or human who cannot adaptively time its expectations and behaviors to distinguish expected vs. unexpected disconfirmations will fail to successfully learn many kinds of behaviors in social settings where timing one’s behaviors to appropriately respond to the behaviors of others is essential for social learning and success. Given the social cognitive problems of some individuals with autism, it is instructive that adaptively timed responses fail to occur in various individuals with autism (Sears et al., 1994; Szelag et al., 2004).

Contextually appropriate timing of motivated responses is, for example, often needed to share *joint attention*, which is often deficient in individuals with autism (Filipek et al., 2000), and to thereby be able to carry out successful imitation learning (Grossberg and Vladusich, 2010), or even to receive action-contingent reward. Moreover, socially unsuccessful behaviors due to bad timing can lead to large numbers of unexpected outcomes, and thus to persistent novelty-sensitive arousal bursts (**Figure 7C**). Section 3.1 will explain how such arousal bursts can, in turn, cause hypersensitive emotional reactions that may lead to coping strategies to prevent these reactions, including an insistence on sameness and circumscribed interests. The persistent failure to get reward may additionally contribute to the development of insufficiently aroused value categories, thereby exacerbating these hypersensitive emotional responses.

#### Spectral Timing and Hippocampal Time Cells

What is the neural mechanism that realizes adaptively timed learning? Adaptively timed learning is carried out by a neural mechanism that is called *spectral timing* (Grossberg and Schmajuk, 1989). Spectral timing enables the START, iSTART, and nSTART models to span an interstimulus interval (ISI), or temporal gap, of 100s of milliseconds, or even seconds, between the offset of a CS and the onset of an US during trace conditioning, or other learning experience with a delayed reward or punishment. Such a delay is orders of magnitude larger than the typical response rates of individual neurons. This learning mechanism is called spectral timing because it activates a “spectrum” of cells which respond at different, but overlapping, times. After this type of adaptively timed learning occurs, the population of these cells, acting together, can generate a population response that is maximal at, or near, the time when the US is expected (Grossberg and Schmajuk, 1989; Grossberg and Merrill, 1992, 1996). This kind of response was originally reported in neurophysiological experiments about adaptively timed conditioning in the hippocampus (Berger and Thompson, 1978; Nowak and Berger, 1992; Tieu et al., 1999).

**FIGURE 10:**
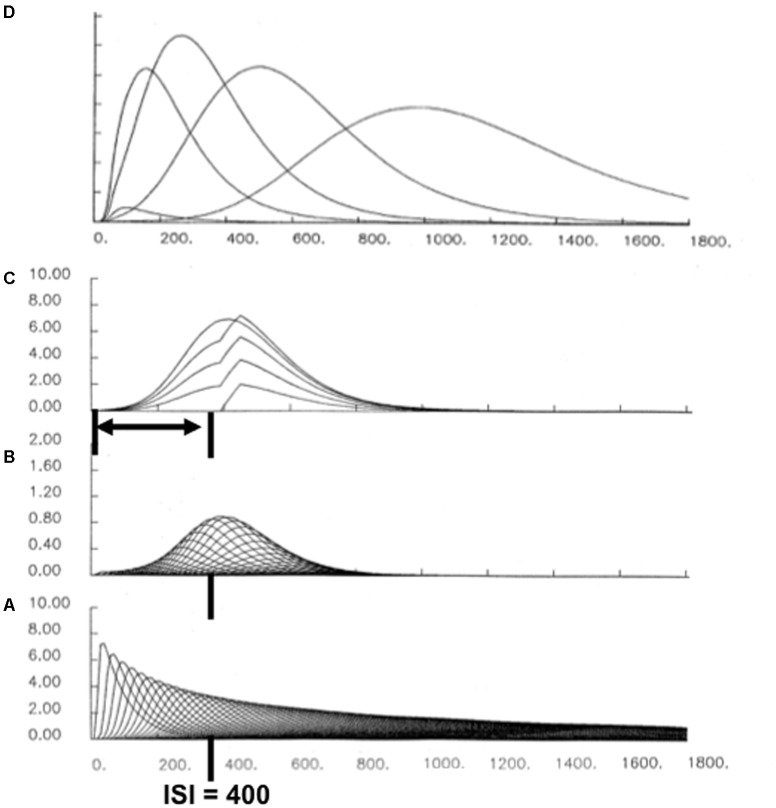
A simulation of adaptively timed learning by a spectral timing circuit: **(A)** A spectrum of cells whose activities respond maximally at different times. **(B)** When a CS and US are paired across learning trials with an ISI of 400 ms, adaptive weights of cells that are active during that time grow proportionally to the activity of their spectral cell. When these weights are multiplied by the spectrum in **(A)**, the resulting learning-gated spectral signals have their largest sizes near the ISI. **(C)** When all the learning-gated spectral signals are added up, the population response peaks at the ISI. The superimposed curves show the growth of the timed response over the first four conditioning trials, followed by a response during recall. **(D)** When the population responses that are learned at different ISIs are all plotted, a Weber-law property obtains, with curves that peak later having broader curves. There is also an Inverted-U of maximal activity, with optimal conditioning occurring at an intermediate ISI, and with learning attenuated at zero and large ISIs.

Each cell in such a spectrum reaches its maximum activity at different times (**Figure 10A**). If the cell response peaks later, then its activity duration is broader in time (**Figure 10A**). This is also true for the adaptively timed population response. **Figure 10D** shows the population responses after learning with different interstimulus intervals, or ISIs. The increase of response variance with ISI is called a *Weber law*, or scalar timing, property (Gibbon, 1977). In addition to generating the Weber law, these model population responses also exhibit the familiar Inverted-U of learning as a function of ISI, with learning attenuated both at very small, and very large, ISIs. Within this range, learned responses are timed to match the statistics of the learning environment (e.g., Smith, 1968).

Recent neurophysiological data about “time cells” in the hippocampus have strongly supported the spectral timing model prediction that a spectrum of cells with different peak activity times obey a Weber law. Indeed, MacDonald et al. (2011) wrote: “…the mean peak firing rate for each time cell occurred at sequential moments, and the overlap among firing periods from even these small ensembles of time cells bridges the entire delay. Notably, the spread of the firing period for each neuron increased with the peak firing time…” (p. 3). MacDonald et al. (2011) have hereby provided direct neurophysiological support for the prediction that spectrally timed cells exist (“small ensembles of time cells”) and that these cells obey a Weber law (“spread of the firing period…increased with the peak firing time”).

The adaptively timed population response is generated by multiplying, or gating, each spectral cell activity by an adaptive weight, or long-term memory (LTM) trace (**Figure 10B**). Each of these LTM-gated cell activities is then added to compute the population response (**Figures 10C,D**). During conditioning, each adaptive weight is amplified or suppressed if its cell activity does, or does not, overlap times when the US occurs; namely, times close to the ISI between CS and US. Learning hereby selectively amplifies output signals from cells whose timing matches the ISI, at least partially (**Figure 10B**). Most cell activity intervals do not match the ISI perfectly. However, the population response that computes the *sum* of the LTM-gated signals from all the cells is well-timed, and typically peaks at or near the expected ISI of the US (**Figures 10C,D**). Spectrally timed learning hereby enables the START model to learn associations between events that are separated in time, notably during trace conditioning.

#### Why a Weber Law? Reconciling Sustained Inhibition of Orienting With Correct Timing

Spectral timing reconciles two potentially incompatible design constraints. On the one hand, the learned spectrum should peak at around the ISI in order to maximize the probability that the behavior is correctly timed. On the other hand, the orienting system should remain inhibited throughout the preceding time interval (**Figure 9**) to prevent an attention shift and maladaptive exploratory behavior before the expected reward occurs. These two constraints are both satisfied because of the Weber law property: Each curve in **Figure 10D** begins to grow at time zero, so it can inhibit the orienting system throughout the initial time interval, but it peaks around the ISI to maximize the probability of a correctly timed response.

#### Multiple mGluR-Modulated Circuits for Timed Learning, Attention, and Action

As noted in Section 2.2 spectral timing has successfully modeled behavioral, neurophysiological, and anatomical data about several parts of the brain: the hippocampus to maintain motivated attention on prefrontal plans for an adaptively timed interval to enable completion of a goal-oriented action (Grossberg and Schmajuk, 1989; Grossberg and Merrill, 1992, 1996; cf., Friedman et al., 2000), the cerebellum to read out adaptively timed movements while motivated attention is maintained (Berger and Thompson, 1978; Ito, 1984; Fiala et al., 1996), and the basal ganglia substantia nigra pars compacta (SNc) to release dopamine bursts and dips. These bursts and dips regulate new associative learning in multiple brain regions in response to unexpectedly timed reward and non-reward (Schultz et al., 1992; Schultz, 1998; Brown et al., 1999, 2004; Gerfen, 2000; Goto and Grace, 2005).

In all of these cases, the metabotropic glutamate receptor (mGluR) system plays a critical role in enabling cell responses to bridge long time intervals. Fiala et al. (1996) have, for example, developed a detailed neural model of the underlying biochemistry of spectral timing in the cerebellum. Fiala et al. (1996) simulated how slow responses may be generated postsynaptically by mGluR-mediated phosphoinositide hydrolysis and calcium release from intracellular stores. These responses are capable of bridging the interstimulus interval (ISI) between the CS-activated parallel fibers that contact Purkinje cells, and the US-activated climbing fibers that deliver teaching signals to the Purkinje cells (**Figure 4**), thereby causing learned long-term depression, or LTD, at (parallel fiber)-(Purkinje cell) synapses.

#### Explaining Fragile X Symptoms Redux

Fragile X symptoms and the role of mGluR dynamics in causing them can now be better understood as a consequence of cognitive, emotional, and behavioral problems that can occur if the adaptively timed circuits that are needed for learning, and consolidating memories of, temporally delayed associations break down. If spectral timing circuits in hippocampus, cerebellum, and basal ganglia are all deficient, say due to inoperative or degraded mGluR dynamics, then all the kinds of data that were summarized in Section 2.3 have immediate mechanistic explanations.

For example, children with Fragile X can exhibit behavioral problems of severe inattention (Fryns et al., 1984; Baumgardner et al., 1995) and ADHD symptoms (Cornish et al., 2004) because their hippocampal adaptively timed circuits cannot maintain motivated attention long enough to successfully carry out many behaviors. The mouse model for FXS experiences severe impairment of trace conditioning (Zhao et al., 2005) for the same reason: its circuit for spectral timing in the hippocampus is not working. Finally, mGluR and FMR1 abnormalities in cerebral cortical and hippocampal synaptic processes can cause deficient cognitive, learning, and motor deficits in Fragile X patients (Huber et al., 2002; Koekkoek et al., 2005; Nosyreva and Huber, 2006; Guo et al., 2012; Vinueza Veloz et al., 2012) because of the several ways in which hippocampal, cerebellum, and basal ganglia spectrally timed circuits support these processes.

If there is a way to pharmacologically restore mGluR dynamics in otherwise intact hippocampal, cerebellar, and basal ganglia circuits, then that could ameliorate FXS symptoms by restoring adaptively timed learning. If not, then operant conditioning methods may be helpful that either differentially reward sustained attention for increasingly long time intervals, or punish orienting behaviors during these time intervals. The net effect will hopefully be the ability to maintain attention for increasingly long time intervals, until it is time to learn a contextually adaptive response.

## 3. Several Causes of Perseverative Behaviors During Normal and Autistic Behaviors

As noted by the American Psychiatric Association (2013), restricted and repetitive patterns of behavior are required for a diagnosis of autism spectrum disorder. Several different brain processes contribute to such behaviors (Baron-Cohen, 1989, 1992; Bodfish et al., 2000; Miller and Neuringer, 2000; Matson and Nebel-Schwalm, 2007; Lam et al., 2008). Some are affective processes that are regulated by brain regions like the amygdala and hypothalamus. Others are motoric processes that are regulated by brain regions like the basal ganglia. The text below proposes mechanistic explanations of several distinct causes for such restricted and repetitive behaviors.

By distinguishing the affective amygdala/hypothalamic mechanisms that contribute to the insistence on sameness and circumscribed interests, from the volitional basal ganglia mechanisms that support stereotyped RMBs, it should become easier to develop targeted therapies to ameliorate these distinct behavioral symptoms. In particular, although operant differential reinforcement of other behaviors (DRO) and differential reinforcement of low rates of responding (DRL) may reduce some stereotyped and self-injurious behaviors that are due to an amygdala/hypothalamic involvement (Gunter et al., 1984; Smith, 1987; Wong et al., 1991; Miller and Neuringer, 2000), they may not directly affect the basal ganglia gating mechanisms that can endogenously generate and maintain other types of RMBs, as the following text will explain.

### 3.1. Amygdala and Hypothalamic Affective Influences on Repetitive Behaviors

#### Opponent Processing in Value Categories: Antagonistic Rebounds

The text will first discuss affective mechanisms that contribute to the insistence on sameness and circumscribed interests. In order to explain how these mechanisms work, the value categories in the amygdala/hypothalamus (**Figure 8**) need to be refined to incorporate circuits that control *opponent* emotional states. After summarizing some main properties of these opponent processes, the text can explain how they can become *underaroused*, When this happens, paradoxical symptoms of emotional unresponsiveness combined with emotional hypersensitivity can be explained.

Value categories in amygdala/hypothalamus contain ON cells and OFF cells that are organized in opponent processes that are called *gated dipoles* (Grossberg, 1972a,b, 1980, 1984; Grossberg and Seidman, 2006; Dranias et al., 2008; Grossberg et al., 2008). These ON and OFF cells can represent opponent emotional and motivational states, such as fear vs. relief, hunger vs. satiety, and so on. Gated dipoles help to explain many data about both classical and operant conditioning, including conditioned acquisition, extinction, learned escape and avoidance, attentional blocking and unblocking, partial reinforcement acquisition effect, gambling behaviors, self-punitive behaviors, and other behavioral properties that currently have no other mechanistic neural explanations. Neurophysiological data from the hypothalamus that match affective gated dipole properties have also been simulated (Dranias et al., 2008; Grossberg et al., 2008).

**FIGURE 11:**
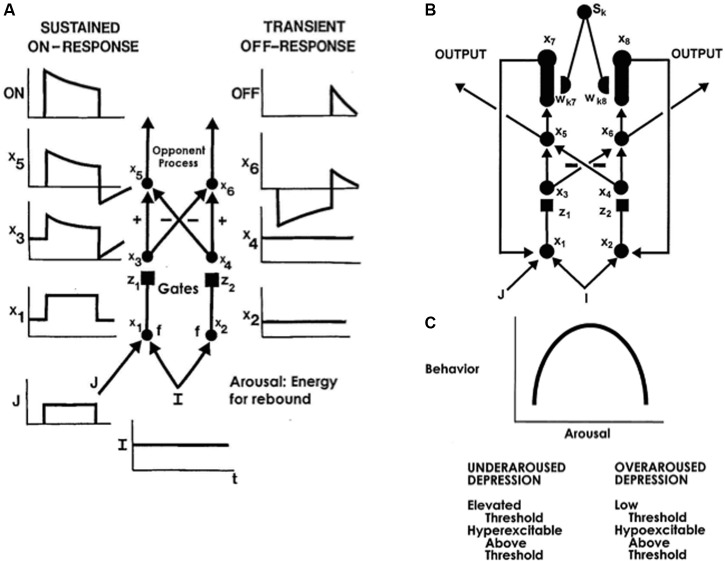
**(A)** A gated dipole opponent process in a value category can generate habituative ON responses and transient OFF rebounds in response to phasic cue onset and offset, respectively. See text for details. **(B)** A READ (REcurrent Associative Dipole) circuit is a gated dipole with excitatory feedback, or recurrent, pathways between activities *x*_7_ and *x*_1_, and activities *x*_8_ and *x*_2_. Sensory representations *S_k_* send∖conditionable signals to the READ circuit that are gated by conditioned reinforcer adaptive weights, or long-term memory (LTM) traces, *w*_*k*7_ and *w*_*k*8_ to the ON and OFF channels, respectively. Read-out of previously learned adaptive weights is dissociated from read-in of new values of the learned weights. This dissociation allows new weight learning to be generated by teaching signals from the ON or OFF channel that wins the opponent competition. The combination of recurrent feedback and associative dissociation enables the adaptive weights to avoid learning baseline noise, while they maintain in short-term memory the relative balance of ON and OFF channel conditioning during a motivated act, and preserve their learned conditioned reinforcer associations until they are disconfirmed by predictive mismatches if and when new learning contingencies are experienced. **(C)** When the tonic arousal level is chosen between low and high values, an Inverted-U in gated dipole responsiveness is caused. At low arousal levels, an underaroused depressive syndrome occurs. At overaroused arousal levels, an overaroused depressive syndrome occurs. See text for details. [Reprinted with permission from Grossberg and Schmajuk (1987).]

The simplest gated dipole circuit is depicted in **Figure 11A** (Grossberg, 1972b). It has non-recurrent, or feedforward, pathways. When gated dipoles augment the dynamics of the CogEM model in **Figure 8**, their hypothalamic ON and OFF channels deliver inputs to the amygdala which, in turn, provides incentive motivational signals to object-value categories in the orbitofrontal cortex, and thereby influences what actions are taken to achieve valued goals. Gated dipoles help to explain how changing reinforcement contingencies alter motivated behaviors because they respond to either sudden decreases in reinforcing inputs, or to unexpected events, with an *antagonistic rebound* that shuts off ongoing ON cell activity and transiently excites OFF cell activity. The transient OFF cell activation is the antagonistic rebound (**Figure 11A**).

For example, a sudden reduction of a fearful shock can cause a relief rebound. Likewise, the non-occurrence of an expected shock can cause a relief rebound (Masterson, 1970; Reynierse and Rizley, 1970; Denny, 1971). The unexpected non-occurrence of food can, in contrast, cause a frustrative rebound (Amsel, 1962, 1992). Thus, rebounds can occur from negative to positive affects, such as from fear to relief, or from positive to negative affects, such as from hunger to frustration. These antagonistic rebounds enable the brain to modify its reinforcement learning to quickly adapt to changing reinforcement contingencies. For example, if the sudden reduction of a fearful shock is due to a successful escape behavior, then the relief rebound can trigger new conditioned reinforcer learning and incentive motivational learning (**Figure 8**), using relief to motivate that escape behavior. In a similar way, the frustrative rebound that occurs after expected food does not occur can drive forgetting, or extinction, of motivational support for the consummatory actions that no longer lead to food.

Simple mechanisms, occurring in a prescribed order, enable gated dipoles to cause antagonistic rebounds either in response to changes in reinforcer amplitude, or to disconfirmations of cognitive expectations of reward. The reader who does not wish to immediately read the mechanistic explanation of how this happens can jump directly to the next section.

These mechanisms are: non-specific arousal (I in **Figure 11A**), cell activation (variables *x_i_* with *i = 1–6* in **Figure 11A**), activity-dependent habituative transmitters (variables *z_i_* with *i = 1* and *2* in **Figure 11A**), competition (pathways with plus and minus signs in **Figure 11A**), and output thresholds (which cause the final ON and OFF cell output signals). The antagonistic rebound in response to offset of a phasic input, such as a shock to the ON channel (variable *J* in **Figure 11A**), is the transient OFF-response (e.g., relief) at the output stage of the OFF channel. This rebound is energized by a tonically active input *I* that delivers arousal equally to both the ON and OFF gated dipole channels (**Figure 11A**).

The ON and OFF cell activities *x*_1_ and *x*_2_ in **Figure 11A** respond to the sum of tonic-plus-phasic ON input *I*+*J*, and the tonic OFF input *I*, respectively, before they generate output signals *f(x*_1_*)* and *f(x*_2_*)* to the next processing stage. Before they reach the next processing stage, these signals are multiplied, or gated, by the habituative transmitters *z*_1_ and *z*_2_, respectively. The gated output signals *f(x*_1_*)z*_1_ and *f(x*_2_*)z*_2_ excite the ON and OFF cell activities *x*_3_ and *x*_4_, respectively, at the next processing stage. The habituative transmitters transform the step-plus-baseline activity pattern *x*_1_ in the ON channel into the overshoot-habituation-undershoot-habituation pattern at activity *x*_3_. The baseline activity pattern *x*_2_ in the OFF chancel is converted into the habituated baseline activity *x*_4_.

Next, the opponent competition occurs across the ON and OFF channels. As a result, the habituated baseline activity *x*_4_ in the OFF channel is subtracted from the ON activity *x*_3_ to compute *x*_5_. The overshoot and undershoot in *x*_5_ are now shifted to be above and below the equilibrium activity zero, respectively. Then activity *x*_5_ is thresholded by half-wave rectification to generate an ON output signal. This output signal has an initial overshoot of activation, after which it habituates. The undershoot is inhibited to zero by the output threshold. The signs of excitation and inhibition are reversed in the OFF channel, leading to activity *x*_6_. Activity *x*_6_ is simply the flipped, or mirror, image of *x*_5_ with respect to the zero equilibrium activity. Thresholding *x*_6_ inhibits to zero the flipped overshoot, while allowing the flipped undershoot to generate the OFF channel output. This is the transient antagonistic rebound. Thus, the antagonistic rebound is due to a combination of arousal, habituative transmitter gating, competition, and thresholding.

The non-recurrent gated dipole must be refined to realize additional properties that are important in the control of learning and behavior. In particular, feedforward interactions are not enough. A *recurrent*, or feedback, gated dipole circuit is needed to realize additional learning properties. The recurrent gated dipole in **Figure 11B** is called a READ circuit, for REcurrent Associative Dipole (Grossberg and Schmajuk, 1989), There is recurrent feedback in both the ON and OFF channels: Activity *x*_7_ reactivates *x*_1_ in the ON channel, while activity *x*_8_ reactivates *x*_2_ in the OFF channel. In addition, adaptive weights, or long-term memory (LTM) traces, *w*_*k*7_ and *w*_*k*8_ sample the ON and OFF channels, respectively, thereby allowing multiple objects and events, with sampling signals *S_k_*, to learn to become conditioned reinforcers when they are associated with reinforcing events at the gated dipole.

A READ circuit can support several basic functional properties (Grossberg and Schmajuk, 1989): First, it can maintain steady motivation while a behavior is being performed, even during sufficiently small environmental distractions, but can rapidly switch to support a new behavior with a different motivation if the distraction is big enough. Second, it enables affective learning to remain sensitive to any number of reinforcing events throughout the lifespan; the LTM traces do not saturate. Third, it enables affective memories to be preserved for a long time, even years, until reward or punishment schedules change, or cognitive expectations are disconfirmed. They can then be quickly modified. Finally, these properties help to explain data about primary and secondary excitatory and inhibitory conditioning, among other important properties.

#### An Affective Inverted-U: Hypersensitive Underaroused Opponent Processes

Arousal typically remains within an optimal range to ensure useful value category properties. Maintaining this optimal range during waking hours is a major achievement of the affective brain. Failure to do so is reflected in behavioral symptoms of several mental disorders, including autism. In particular, the activity of a gated dipole circuit exhibits an Inverted-U as a function of its tonic arousal level *I* (**Figure 11C**; Grossberg, 1984; Grossberg and Seidman, 2006): Gated dipole outputs in response to either abnormally small or abnormally large arousal inputs are depressed. Intermediate arousal input sizes generate a Golden Mean of responding at the middle of the Inverted-U. These gated dipole properties are a consequence of the same mechanisms that enable a gated dipole to trigger antagonistic rebounds and to thereby quickly adapt to changing reinforcement contingencies. In particular, the Inverted-U can be traced to how the state of habituation in the dipole’s transmitter gates (square synapses in **Figures 11A,B**) *divide* the effects of signals through the dipole. This division creates a Weber Law of dipole responsiveness.

In an underaroused gated dipole, the response threshold to inputs is abnormally high but, after input intensity exceeds this elevated threshold, further increments in input intensity lead to *hypersensitive* emotional responses (**Figure 11C**). This happens because the habituative transmitter that divides dipole responses in abnormally small. These properties help to explain why individuals with autism may show at best small affective responses to some inputs, but hypersensitive responses to inputs that exceed the elevated threshold. This property can interact with the hypervigilance of some individuals with autism to cause emotional outbursts in situations where individuals without autism would not respond in this way.

As noted in Section 2.4 hypervigilant individuals learn hyperconcrete recognition categories that are accompanied by a narrow focus of attention. Many events that would not be unexpected to a person with a broader and more flexible attentional focus are unexpected to an hypervigilant individual, thereby causing multiple arousal bursts when environments change even moderately (**Figure 7C**). These arousal bursts, in turn, can cause hypersensitive emotional reactions if they input to underaroused hypothalamic gated dipoles. The frequent hypersensitive emotional responses when environments change can be so aversive that individuals with autism may learn to avoid them by indulging in a reduced set of behaviors, including perseverative behaviors that seek to maintain a level of sameness which avoids the mismatches that would otherwise trigger hypersensitive emotional responses.

#### Insistence on Sameness and Circumscribed Interests: How Operant Conditioning Helps

With this background in hand, one can begin to understand how an individual with autism may seek refuge in an insistence on sameness and circumscribed interests.

Because this mechanism for shaping a need for sameness and restricted interests involves value categories that regulate reinforcement learning and motivated attention (**Figure 8**), it can be modified by operant reinforcement contingencies that differentially reward variable behaviors (e.g., Miller and Neuringer, 2000; Lam et al., 2008), and thereby strengthen their conditioned reinforcer and incentive motivational pathways so that they can competitively inhibit pathways that support more restricted behaviors.

These operant manipulations may not, however, directly modify the basal ganglia circuit properties that can support RMBs of individuals with autism. How these behaviors may be caused is explained in Section 3.2.

### 3.2. From Normal Basal Ganglia Motor Gating to Repetitive Behaviors in Autism

In addition to the role of the substantia nigra pars compacta (SNc) in generating widespread dopaminergic Now Print signals to support new associative learning (see Section 2.2), the basal ganglia also control the opening and closing of gates in the substantia nigra pars reticulata (SNr) that enable cognitive and motor processes to be carried out [**Figure 2**; see Grossberg (2016) for a review]. In particular, neural models have explained how opening an SNr basal ganglia gate can release an action whose properties are controlled by downstream circuits (e.g., Bullock and Grossberg, 1988; Brown et al., 1999, 2004; Grossberg and Paine, 2000; Grossberg and Pearson, 2008; Silver et al., 2011). Normally, such gating events are under volitional control. Sustained opening of a gate can sometimes elicit repetitive behaviors that are controlled by recurrent, or feedback, circuits that are downstream from the gate. The recurrent circuit responds to gate opening by generating oscillatory dynamics that cause the same behavior to occur, over and over again. Section 3.3 summarizes how the basal ganglia can cause repetitive behaviors in normal individuals in this way. This background clarifies that the machinery for repetitive behaviors exists in all brains. Sections 3.4 and 3.5 explain how repetitive behaviors can be caused in individuals who are confined within restricted spaces. Here, circadian and motivational mechanisms again play an important role. Section 3.6 explains how RMBs in individuals with autism (Turner, 1999; Bodfish et al., 2000; Militerni et al., 2002; McBride, 2015) may be generated by imbalances in the direct and indirect pathways of the basal ganglia that keep gates open long enough for oscillatory recurrent networks further downstream to maintain RMBs that may not be under volitional control.

### 3.3. Normal Repetitive Behaviors: Motor Gaits and Saccade Staircases

Normal perseverative behaviors include repetitive motor gaits, such as walking or running (Brown, 1911), and saccade staircases, or series of stereotyped saccadic eye movements, that are caused by sustained electrical stimulation of the superior colliculus (Schiller and Stryker, 1972). Neither of these behaviors is always perseverative: Sustained postures such as standing or sitting typically alternate with walking or running, and individual saccades are the norm, not saccade staircases.

**FIGURE 12:**
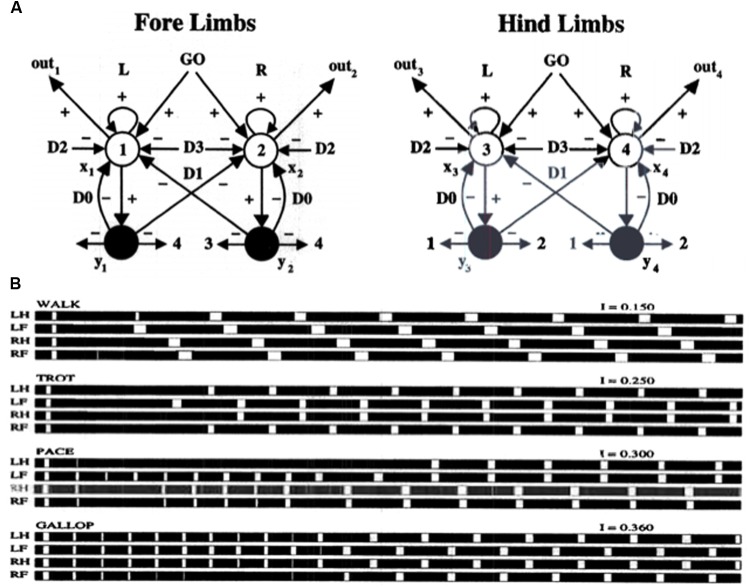
A central pattern generator, or CPG, that is capable of generating well-known series of movement gaits using a recurrent on-center ( + signs) off-surround ( – signs) network whose cells obey the membrane equations of neurophysiology (shunting interactions) when it is activated by a GO signal of variable size: **(A)** The network is defined by a four-channel oscillator. Inhibitory connections between the forelimbs and hindlimbs are represented by arrows originating at the source of the inhibition and numbered by the label of the cell that is the destination. A like-labeled arrow represents the destination of this inhibition. The network has self-inhibition labeled by the parameter D0, inhibition between forelimbs and between hindlimbs labeled by D1, inhibition between matched forelimbs and hindlimbs labeled by D2, and connections between crossed forelimbs and hindlimbs labeled by D3. **(B)** Computer simulation of how an increasing GO signal, along with GO-modulated modulation of the inhibitory coefficients, yields an ordered series of gaits (walk, trot, pace, and gallop) as an emergent property of network interactions. [Reprinted with permission from Pribe et al., 1997).]

Both of these repetitive behaviors may be traced to prolonged opening of an appropriate basal ganglia SNr gate, or equivalent event, throughout the repetitive performance. During gaits, opening an appropriate gate has the effect of turning on a GO signal that drives a central pattern generator, or CPG, in the spinal cord (**Figure 12A**). A neural model of CPG dynamics generates gaits and the observed transitions between them (Pribe et al., 1997). This CPG is a specialized recurrent on-center off-surround network whose cells obey the membrane equations of neurophysiology (**Figure 12A**; Ellias and Grossberg, 1975), otherwise called shunting interactions. Increasing the model’s volitional GO signal causes the CPG to transition from one gait pattern to another (**Figure 12B**), thereby simulating gait transitions in cat (walk-trot-pace-gallop), human (walk-run), and elephant (amble-walk) in variants of this CPG circuit.

**FIGURE 13:**
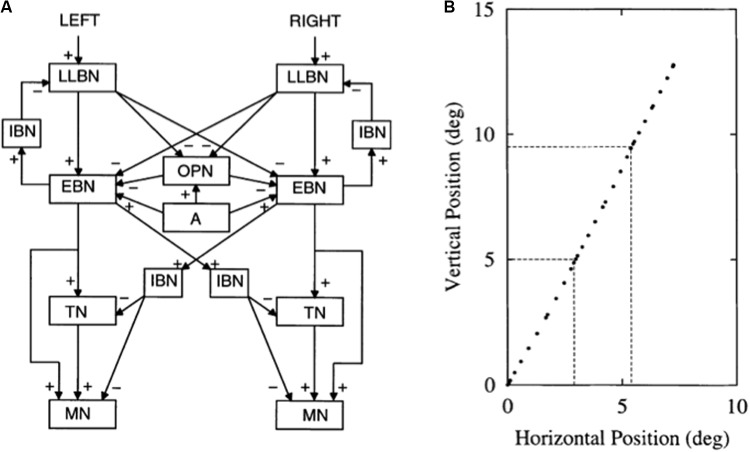
The FOVEATE (Feedback Opponent VEctor ArchiTEcture) neural model of the saccade generator circuit in the peripontine reticular formation for control of an antagonistic pair of extraocular muscles. **(A)** Note the recurrent interactions between long-lead burst neurons (LLBN), excitatory burst neurons (EBN), and inhibitory burst neurons (IBN). These recurrent interactions support saccade staircases when movement gates (omnipause neurons) stay open long enough. Omnipause neurons = OPN; arousal signal = A; tonic neurons = TN; and motorneurons = MN. **(B)** Computer simulation of a sequence of three saccades, all in the same direction as the initial saccade, that is caused by a sustained constant input to the saccade generator. Eye position was sampled at regular time intervals. Reprinted from Gancarz and Grossberg (1998).]

The basal ganglia also control the release of ballistic eye movements called saccades (Hikosaka and Wurtz, 1983; Kori et al., 1995; Handel and Glimcher, 1999, 2000; Shaikh et al., 2011). In a normal brain, the basal ganglia SNr tonically inhibits the deeper layers of superior colliculus (SC). When this inhibition is disinhibited by gate opening at a particular collicular position, a saccade can be elicited in the direction and distance that is represented by that position. The commanded saccadic direction and distance are converted into a saccade with those parameters by a recurrent circuit within the peripontine reticular formation. The FOVEATE (Feedback Opponent VEctor ArchiTEcture) neural model (**Figure 13A**; Gancarz and Grossberg, 1998) models this reticular formation recurrent circuit, and simulates behavioral and neurobiological data about how it generates saccadic eye movements.

Sustained electrical stimulation to the deeper layers of the SC has an effect equivalent to keeping a basal ganglia gate open for an unusually long time, resulting in a series of saccades of the same amplitude and duration; that is, a *saccade staircase* (Schiller and Stryker, 1972; McIlwain, 1986). Saccade staircases are also generated by the FOVEATE model when the gate remains open for a long enough time (**Figure 13B**).

The above examples show how, even in normal individuals, sustained opening of an SNr gate can trigger repetitive behaviors that are controlled by recurrent neural circuits that are downstream from the open gate.

### 3.4. Repetitive Behaviors in Restricted Environments: Tonic Exploratory Drive

Repetitive behaviors also occur in normal animals who are housed in restricted environments, such as in a zoo, farm, and laboratory (Mason, 1991; Wurbel, 2001), or who experience early social deprivation (Harlow et al., 1965). Unlike repetitive behaviors that are caused when a phasically active basal ganglia gate stays open for too long, these repetitive behaviors may be traced to tonically active GO signals that energize the exploratory behaviors which enable terrestrial animals to find food and other necessities, but which are prevented in restricted environments. These sustained GO signals are, in turn, energized by output signals of a circadian pacemaker in the suprachiasmatic nuclei (SCN) of the hypothalamus (Stephan and Nunez, 1977). These signals provide a critical component of the arousal that energizes the hypothalamic affective gated dipoles that form part of the value categories of the CogEM model (**Figures 8**, **11**).

### 3.5. Circadian and Generalized Drive Effects on Motivated Behaviors

**FIGURE 14:**
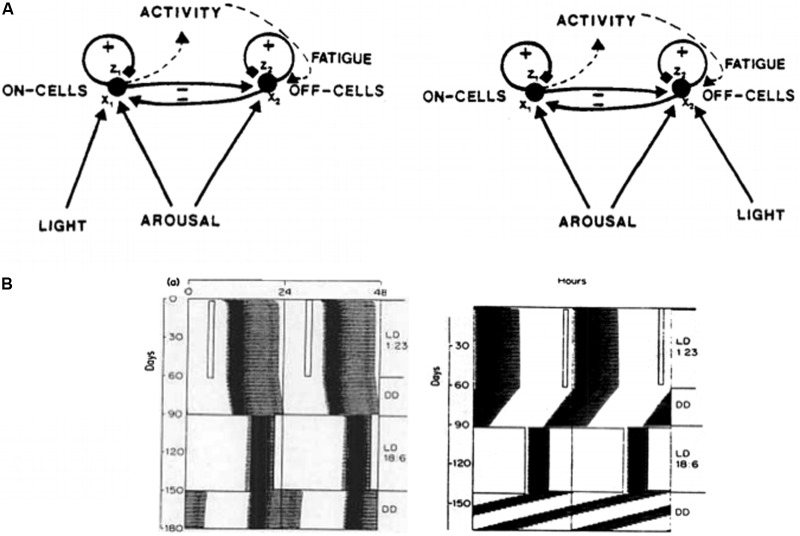
**(A)** Diurnal and nocturnal gated pacemaker circuits of the circadian oscillator in the suprachiasmatic nuclei (SCN). In both of these recurrent circuits, ON cells and OFF cells excite themselves via positive feedback, inhibit each other via negative feedback, and are tonically aroused. Light excites ON cells in the diurnal circuit and OFF cells in the nocturnal circuit. Activation of ON cells or suppression of OFF cells energizes wakefulness and activity. Fatigue builds up during the wakeful state and excites OFF cells in both diurnal and nocturnal circuits. **(B)** Two computer simulations of photoperiod after-effects. In both simulations, the model is exposed to a Light-Dark (LD) 1:23 lighting regime (1 h of light every 24 h) before free-running in the dark. Then the model experiences a LD 18:6 lighting regime before free-running in the dark. The free-running activity levels and periods depend upon the prior lighting regimes and persist through the 30-day free-run intervals. Each figure is a double-plot. Two successive days are plotted in each row and each successive day is plotted in the left-hand column. Thus the day plotted in the right-hand column of the i^th^ row is also plotted in the left-hand column of the (i + 1)^st^ row. [Reprinted with permission from Carpenter and Grossberg (1985).]

Carpenter and Grossberg (1983, 1984, 1985) have modeled circadian activity cycles using a *gated pacemaker* neural model of SCN dynamics (**Figure 14A**). Butler et al. (2012) summarize recent data that support this SCN model. The SCN pacemaker supports goal-oriented operant behaviors that can fill an animal’s waking hours (**Figure 14B**), with specific behaviors that may be energized by incentive motivational signals from active value categories (**Figure 8**), when they are aroused by the circadian pacemaker in the SCN (**Figure 14B**; Abrahamson et al., 2001).

There are many homologous circuit elements in the SCN gated pacemaker and the gated dipoles of value categories. Given their anatomical proximity, it is tempting to predict that they are variations of a shared circuit design. For starters, both are opponent processes with ON and OFF cells. The gates in the gated pacemaker model are habituative transmitters, just as in a gated dipole value category (**Figures 11**, **14**). In the circadian clock circuit, the opponent process is a recurrent on-center off-surround network whose circadian oscillation is energized by a tonically active arousal level, just as in a gated dipole value category. Just as habituative gating parameters in a hypothalamic eating circuit can determine the time course of eating behaviors, habituative gating parameters in the SCN circadian circuit can determine the period of the circadian clock in the dark. Just as an external food cue can energize the ON channel of a hypothalamic gated dipole eating circuit to trigger eating behavior, a light cue can energize an SCN diurnal gated dipole circuit to trigger operant exploratory behavior (**Figure 14A**). Just as a satiety signal to the off-channel of a hypothalamic gated dipole eating circuit can inhibit eating behavior, a fatigue signal to the off-channel of an SCN gated dipole can inhibit operant behavior (**Figure 14A**). The hypothalamic SCN circadian clock design is thus strikingly similar to that of the nearby amygdala/hypothalamic gated dipole value category circuits of the CogEM model that it arouses.

In a restricted environment, the persistently active GO signals from the SCN cannot be fully expressed and fatigued by the normal exploratory behaviors that they ordinarily support. Instead, they can energize the kinds of behaviors that *are* possible in these environments, much as “generalized drives” can transfer from one operant activity to another under certain conditions (Miller, 1948; Amsel and Maltzman, 1950). Due to the tonic nature of the SCN activation, and the slow time scale of the fatigue signal, the gates that control these possible behaviors can remain open for longer than is normally the case, thereby causing persistent repetitions of them. This can occur because both circadian and internal drive inputs combine with reinforcing cue inputs at a value category such as the amygdala/hypothalamus (**Figure 8**), thereby activating incentive motivational signals to object-value categories in the orbitofrontal cortex and supporting the corresponding operant behavior. At the same time, the amygdala value category helps to activate the nucleus accumbens (NAc; **Figure 2**) and, through it, the corresponding basal ganglia GO signal gates that allow the behavior to be expressed (Friedman et al., 2002; Groenewegen, 2003; Voorn et al., 2004). See below for further discussion of this latter point.

### 3.6. Factors Leading to Prolonged Gate Opening in Individuals With Autism

The examples above show how sustained opening of an SNr gate can elicit a repetitive behavior even in normal individuals when the open gate maintains activation of a recurrent circuit downstream. It will now be shown how such sustained gate opening can occur in individuals with autism as a result of *enhanced activation of the basal ganglia direct pathway or suppression of its indirect pathway* (**Figures 2**, **6**), thereby again enabling recurrent networks further downstream to generate repetitive behaviors. Some compatible data include the following.

#### Influences of Direct and Indirect Pathways on Stereotypy

A study of deer mice with induced stereotypy reported an imbalance in neuronal activity that was expressed in terms of cytochrome oxidase (CO) levels within the motor cortex, striatum, nucleus accumbens, thalamus, and hippocampus. In particular, Lewis et al. (2007) found that animals with high stereotypy rates had low CO levels in these brain regions, while animals with low stereotypy rates showed high CO levels.

Many additional studies on stereotypy are consistent with the proposal that imbalances in the direct and indirect pathway can support repetitive behaviors. For example, it is known that changes in the development of the striatum, where the direct and indirect pathways occur, are involved in repetitive behavior in autism (Langen et al., 2014). Moreover, drug-induced stereotypy manipulations in the SNr of the direct pathway and the sub-thalamic nucleus (STN) of the indirect pathway can cause repetitive behaviors. In particular, administering an intranigral GABA agonist causes stereotypy in rats (Scheel-Kruger et al., 1978), whereas administering a serotonergic (5-HT2) antagonist in the STN reduces stereotypy. These procedures altered either directly (intranigral GABA agonist administration) or indirectly (intra-STN 5HT2 antagonist administration) inhibitory GABAergic tone in thalamocortical relay neurons (Brunken and Jin, 1993). Manipulations that disinhibited thalamocortical projections induced stereotypy, whereas manipulations that inhibited thalamus reduced stereotypy (Barwick et al., 2000). For a similar reason, injecting opiate agonists into the substantia nigra leads to stereotypies in rats (Iwamoto and Way, 1977) by disinhibiting nigrostriatal dopaminergic projections, and thereby presumably elevating striatal dopamine release, as has also been shown in mice (Wood and Richard, 1982).

A more recent study observed that decreased indirect pathway activity occurs in animals that develop high rates of stereotypy (Tanimura et al., 2011). An alternative mechanism involving the balance of striosomal activity and matrix activity within the striatum has been suggested to impact the regulation of behavioral sequences, such that the relative enhancement of striosome-over-matrix activation can predict the amount of stereotypy that develops in the animals, where the ratio of activation was assessed using Immediate Early Gene (IEG) expression (Graybiel et al., 2000). The above experimental studies are thus compatible with the hypothesis that imbalanced basal ganglia circuitry can give rise to stereotypical behavior by allowing prolonged gate opening to release repetitive behaviors from recurrent networks further downstream.

The basal ganglia have also been predicted to regulate action sequences through interactions of the direct, indirect pathways and hyperdirect pathways (Gerfen and Bolam, 2010; Haynes and Haber, 2013; Jin et al., 2014). **Figure 2** illustrates this interaction between the different pathways and how GO and STOP gating signals may be incorporated into this interaction. The direct pathway consists of GABAergic projections from the dorsal striatum to the globus pallidus (GPi) of the SNr, which in turn sends GABAergic neurons to the thalamus. The indirect pathway, on the other hand, consists of GABAergic projections to the external globus pallidus (GPe), which further inhibits the GPi (Graybiel, 2000; Gerfen and Bolam, 2010). Thus, activation of the striatum at the initiation of an action would generate a GO signal that would inhibit the GPi, further disinhibiting the thalamus and permitting the release of action sequences. Activating the dorsal striatum at the end of the action sequence would initiate a STOP signal in the indirect pathway, which disinhibits the GPi, thus counteracting the action of the GO signal.

#### Model Simulations of Neural Control of Movements and Movement Sequences

The non-execution of a plan or action has been attributed in modeling studies to either the inability to activate a sufficiently strong GO signal, or an overactive STOP signal blocking the implementation of the plan, or some combination of these GO and STOP signals acting together. In particular, Brown et al. (2004) have supported these claims about GO and STOP basal ganglia dynamics with explanations and simulations using their TELOS neural model (**Figure 6**). TELOS simulates how monkeys learn five saccadic eye movement tasks (fixation, saccade, overlap, gap, and delayed) using interactions between the basal ganglia; prestriate, inferotemporal, parietal, and prefrontal cortices; frontal eye fields; and superior colliculus. After learning of all five movement tasks, TELOS was able to use the learned parameters to simulate the neurophysiologically recorded dynamics of 17 different types of identified neurons during these behaviors. Silver et al. (2011) extended TELOS to the lisTELOS model to simulate learning and performance of *sequences* of saccadic eye movements from a spatial working memory, guided by the temporally coordinated firing of three different basal ganglia loops, and to simulate challenging neurophysiological data about this kind of task.

In addition to modeling studies that clarify how the direct and indirect pathways interact, it is known that the subthalamic nucleus (STN), which comprises part of the cortico–STN–pallidal hyperdirect pathway, has glutamergic projections to both the GPi/SNr and GPe, and hence has the ability to influence both direct and indirect pathways (Aron and Poldrack, 1999; Charpier et al., 2010). Nambu et al. (2002) describe how an engaged motor program is initiated, executed, and terminated with the correct timing, while other competing programs are inhibited. In particular, just before a voluntary movement is initiated, a signal through the cortico–subthalamo–pallidal *hyperdirect* pathway inhibits large areas of the thalamus and cerebral cortex that are related to, and that would otherwise compete with, the selected motor program. Then a second signal through the cortico–striato–pallidal *direct* pathway disinhibits and releases the selected motor program. A third signal that engages the cortico–striato–(external pallido)–subthalamo–(internal pallidal) *indirect* pathway inhibits its targets.

#### D1 and D2 Receptors and Nucleus Accumbens for Direct and Indirect Pathway Control

The excitatory and inhibitory actions of the direct and indirect pathways are realized by different receptor types. In particular, the D1 dopamine receptor is expressed mainly by neurons in the direct pathway, while the D2 dopamine receptor is expressed mainly by neurons of the indirect pathway (Graybiel et al., 2000; Gerfen and Bolam, 2010). The D1 receptors have an excitatory effect on the direct pathway, while the D2 receptors have an inhibitory effect on the indirect pathway (Wichmann and DeLong, 1996), as can be seen in **Figure 2**.

Another important region is the nucleus accumbens (NAc), which is a part of the ventral striatum of the basal ganglia that is significant for reward processing (**Figure 2**; Kelley et al., 1997; Knutson et al., 2001). The NAc is also known to have GABAergic projections to the globus pallidus (Swanson and Cowan, 1975; Mogenson et al., 1980; Newman and Winan, 1980). An increase in the inhibitory activity from the NAc to the GPi could reduce the effect of the STOP signal, leading to perseverative behavior through the action of downstream recurrent circuits. In other words, an increase in inhibition of the GPi by the NAc could be one of the causes for an imbalance between direct and indirect pathways that can support perseverative behaviors by enabling a basal ganglia gate to remain open longer than is necessary for eliciting an individual behavioral response.

The following experimental data are relevant to this hypothesis. An imaging study using MRI and other imaging techniques studied the shape of the basal ganglia in boys with autism, comparing them to a control group. They observed an overgrowth of the nucleus accumbens in the form of an outward deformation, in the group consisting of boys with autism. They also observed that the volume of this overgrowth was positively correlated with greater social and communication deficits (Qiu et al., 2010). Mogenson et al. (1980) proposed a model for initiation of locomotor action comprising the caudate nucleus, ventral tegmental area, nucleus NAc, and the GP. These authors summarized experiments showing that the NAc region in mice receives inputs from both the hippocampus and the prefrontal cortex (PFC). They noted that the hippocampus mediates transmission through D1 medium spiny neurons (MSNs), while the PFC mediates transmission occurs through D2 MSNs. If this is the case, then an abnormality in one or both types of information processing could shift the balance of activation in the NAc.

A study of Neuroligin-3 (NL3) mutated mice, who exhibit autism spectrum disorder symptoms, led to the discovery that the D1 MSNs in the NAc show reduced synaptic inhibition compared to excitation (Rothwell et al., 2014). Since the NL3 mutation has been found to produce autism-like symptoms, it is a possible animal model for aspects of autism (Radyushkin et al., 2009). The altered balance of excitation and inhibition between the cortical and limbic pathways could result in over-activity of the NAc, inducing increased GABAergic inhibition of the GP. This increased inhibition would disinhibit the thalamus longer than is required, resulting in the basal ganglia gate being open for a prolonged time, thereby enabling repetition of the gated behavior. The fact that dopaminergic circuits in the basal ganglia may contribute to repetitive behaviors in autism is consistent with the efficacy of dopaminergic, serotonergic, and opiate drugs in diminishing repetitive behaviors in animal models (Lewis and Bodfish, 1998).

Such repetitive behaviors can also be involved in perpetuating a vicious cycle. For example, although a repetitive behavior may help to satisfy the need for sameness by focusing attention on the repeated behavior, by the same token, a repetitive behavior can prevent attention from being focused on reinforcing or socially important sensory cues, and can thereby prevent the kind of recognition learning and social cognitive learning that might help to overcome some of the social isolation that the repetitive behavior perpetuates (cf. Bodfish et al., 2000; Miller and Neuringer, 2000; Lam et al., 2008).

## 4. Conclusion

The article proposes how quantitative neural models of normal behaviors can generate symptoms of FXS and autistic repetitive behaviors when their mechanisms become imbalanced in prescribed ways. In the case of FXS, a host of cognitive, emotional, and behavioral problems can occur when mGluR-modulated adaptive timing mechanisms fail in the hippocampus, cerebellum, and basal ganglia.

Stereotyped behaviors of individuals with autism can take several forms. Some problems can be traced to how hyperconcrete cortical recognition categories and their consequently narrow attentional foci interact with underaroused hypothalamic and amygdala circuits to generate hypersensitive emotional reactions. Coping with these aversive emotional experiences can lead to an insistence on sameness and circumscribed interests. These behaviors may be modified by operant conditioning methods.

Various RMBs may occur when imbalances in the direct and indirect pathways of the basal ganglia keep movement gates open for too long, thereby releasing recurrent circuit oscillations further downstream that are not under volitional control. Therapies that directly or indirectly augmented D2 dopamine receptor responses, or reduced D1 dopamine receptor responses may be helpful. Repetitive behaviors like walking or saccade staircases have a similar explanation in terms of basal ganglia gates kept open, in the former case by volitionally controlled signals to the basal ganglia, and in the latter case by sustained electrode stimulation of the superior colliculus.

Repetitive behaviors in restricted environments have an explanation in terms of interactions between circadian and appetitive midbrain circuits, and thus are also susceptible to modification with operant conditioning techniques.

The explanation of RMBs calls attention to the fact that *all* iSTART circuit mechanisms are part of cortico-striatal loops (Alexander et al., 1986; Alexander and Crutcher, 1990; Grahn et al., 2009) and are thus also subject to basal ganglia gating. Among the open questions for future experimental and modeling research is whether, and to what extent, the kinds of imbalances in basal ganglia gating that influence autistic RMBs also contribute to the cognitive, emotional, and timing symptoms of individuals with autism that iSTART has already explained using circuits that do not include imbalances within the basal ganglia. Such results may demonstrate closer mechanistic links between the basal ganglia imbalances that induce repetitive behaviors and other behavioral symptoms of autism.

## Author Contributions

DK and SG both contributed significantly to the research that is reported in this article. SG did most of the writing of the article.

## Conflict of Interest Statement

The authors declare that the research was conducted in the absence of any commercial or financial relationships that could be construed as a potential conflict of interest.
